# Beneficial effects of melatonin on canine oocyte nuclear maturation via reduction of oxidative stress

**DOI:** 10.1530/REP-24-0388

**Published:** 2025-03-14

**Authors:** Fataneh Ghafari, Mazdak Salavati, Richard J Piercy, Ali A Fouladi-Nashta

**Affiliations:** ^1^ Departments of Comparative Biomedical Sciences, The Royal Veterinary College, Hawkshead Campus, London, UK; ^2^ Department of Clinical Science and Services, The Royal Veterinary College, Hawkshead Campus, London, UK

**Keywords:** melatonin, dog, oocyte, nuclear maturation, oxidative stress, MTNR

## Abstract

**In brief:**

Oxidative stress due to high-fat content damages canine oocytes during long period of *in vitro* maturation and compromises their developmental competence. This paper shows how melatonin protects the oocytes and increases development to metaphase II stage. Results from this study would be applicable to conservation of endangered and other animal species.

**Abstract:**

Unlike other domesticated animals, *in vitro* maturation of canine oocytes results in poor nuclear maturation to the metaphase II stage and high oocyte degeneration. The high-fat content of canine oocytes is the likely cause; it predisposes them to oxidative stress and deleterious reactive oxygen species (ROS). Melatonin (MTN) (potentially acting as a powerful antioxidant) was reported to support *in vitro* cultured oocytes in other species. In this work, canine cumulus oocyte complexes (COCs) were collected after routine ovariohysterectomy. Immunocytochemistry for expression of melatonin receptors (MTNR-1A and 1B) revealed that both receptors were highly expressed in canine oocytes and there was lower expression in the cumulus cells. Canine COCs matured *in vitro* in melatonin-supplemented culture at 100 nM concentration in low (5%) O_2_ incubator had a lower percentage at GV stage (6.7% ± 4.2 vs 19.8% ± 3), a higher MII stage (32.3% ± 6.4 vs 15.81% ± 8.1), lower degeneration (20.5% ± 3.2 vs 45.2% ± 5.15) and higher meiotic resumption (GVBD-MII; 56.2% ± 8.6 vs 19% ± 3), and produced lower ROS (determined after DCHFDA staining) (all *P* < 0.005) than oocytes cultured in high O_2_ (20%) incubator. The expression of ROS-regulating genes *GPX-1* and catalase was significantly reduced in oocytes cultured with melatonin in low O_2_ compared with high O_2_ (*P* < 0.05). Importantly, the oocyte maturation rate increased significantly when G-IVF PLUS, a commercial culture medium used in human oocyte culture, was supplemented with MTN (*P* < 0.05). These results support the main objective of this study to analyze the beneficial effects of melatonin and low oxygen tension on both health and the developmental competence of *in vitro* matured canine oocytes.

## Introduction

In contrast to most mammalian species, canine oocytes are ovulated immature at the prophase of the first meiotic division (germinal vesicle, GV) and complete the maturation process to metaphase II stage (MII) 48–72 h after ovulation in the oviduct (Hewitt & England 1999, De los Reyes *et al.* 2011). *In vitro* maturation (IVM) of canine oocytes is often unsuccessful; in particular, low success rates are characterized by poor nuclear development to MII stage and consequently, poor embryo formation after *in vitro* fertilization. High degeneration (>50%) and very low MII maturation rates (16.2 ± 4.2%) (Luvoni *et al.* 2005) are the main features of IVM in the dog. This has been attributed to the high-fat content of canine oocytes and the source of oocytes used for IVM. Dog oocytes contain abundant lipid droplets that occupy 80–90% of the ooplasm (Songsasen *et al.* 2009). The lipid originates from the precursors of lipid yolk deposits which are transported from follicle cells through the zona pellucida (ZP) (Guraya 1965). Initially, these lipids are detectable in the primary oocyte of growing follicles, the fat content increases throughout oogenesis.

In mammalian tissues, reactive oxygen species (ROS) are produced as byproducts of aerobic metabolism. Low level of ROS production is necessary for gene expression (Allen & Tresini 2000), cell signaling (Khan & Wilson 1995) and redox homeostasis. Maintaining the balance between the generation and elimination of ROS is critical for normal metabolic function in mammals. When ROS production overwhelms the scavenging ability of antioxidants, oxidative stress (OS) occurs, which exerts very toxic effects on cell function and survival. ROS repairing enzymes prevent oxidative damage by interrupting free radical-associated chain reactions. Within the reproductive system, presence of antioxidant enzymes has been reported in ovaries and the reproductive tract (Sugino 2005). These include catalase, superoxide dismutase (SOD), glutathione peroxidase (GPX-1) and glutathione reductase (GR) (Salavati *et al.* 2012). It is noteworthy that hydrogen peroxide (H_2_O_2_) is a byproduct of the ROS protection system, in which SOD enzymes transform damaging oxygen-free radicals into less aggressive H_2_O_2_ molecules (Whitaker & Knight 2008).

The main source of canine oocytes for research is the ovaries of pet dogs that undergo routine neutering (ovariohysterectomy) during anestrus. In the absence of visible ovulatory follicle, the oocytes are retrieved through slicing/chopping of the ovarian cortex. Such invasive mechanical extraction may inflict physical damage to the oocyte or the cumulus cells surrounding the oocyte.

Critically important to consider is the fact that dog oocytes used for IVM are derived mainly from early antral follicles. These oocytes are deficient in vital cytoplasmic components including mRNAs, proteins, signaling molecules, etc., which are essential for competency of the oocyte to complete nuclear maturation (Khan *et al.* 2021). In addition, the insufficient repair mechanisms in oocytes derived from the growing follicles predisposes them to lipid peroxidation ROS generation and OS (Lin *et al.* 2014). Therefore, *in vitro* cultured dog oocytes are exposed to both physical stress caused by the extraction method, and OS due to high-fat content (Guerin *et al.* 2001) after extraction during the prolonged period of 72 h IVM. In addition, unfavorable culture conditions such as high oxygen concentration can also predispose the oocytes to OS and generation of high ROS level, causing oocytes degeneration, failure to resume meiosis or development to MII stage (Rodrigues & Rodrigues 2003, Songsasen & Wildt 2007, Salavati *et al.* 2012, Songsasen *et al.* 2017). Therefore, reduction of OS can profoundly contribute to improved nuclear and cytoplasmic maturation (Guerin *et al.* 2001, Menezo *et al.* 2010).

Overall, the interplay between the source of oocytes used for IVM, high-lipid content of canine oocytes, OS and the limitations of current IVM are vital factors which contribute to low maturation rate. We have previously shown that culture of canine oocytes in hypoxic conditions improves nuclear maturation and reduces oocyte degeneration (Salavati *et al.* 2012). Furthermore, addition of various exogenous antioxidants to culture media improves nuclear maturation, viability and developmental competence of oocytes in several species (Prasad *et al.* 2016, Hardy *et al.* 2021). These antioxidants are associated with maintaining physiological levels of ROS and protecting the oocyte from OS (Rodrigues & Rodrigues 2010, Salavati *et al.* 2012, Moawad *et al.* 2021).

Melatonin (MTN) is an indolamine hormone secreted from the pineal gland and ovaries (Adriaens *et al.* 2006) in mammals. Given its amphiphilic nature, MTN can easily penetrate biological membranes and in so doing, it increases cell membrane fluidity. It can also enter all subcellular compartments by diffusion or via selected receptor binding (Albarracin *et al.* 2005, Winiarska *et al.* 2006, Hardeland *et al.* 2011, Lu & Marti 2020, Artime-Naveda *et al.* 2023). MTN has varied effects on tissues and cells, depending on its source and cell-specific receptor expression, and with its potent antioxidant effects, it protects enzymes, proteins, lipids and DNA from oxidative damage (Grant *et al.* 2009, Janjetovic *et al.* 2017). It promotes expression of ROS repairing enzymes (Lu & Marti 2020), upregulates the synthesis of the antioxidant amino acid glutathione (GSH) (Urata *et al.* 1999) and neutralizes nitric oxide and the peroxynitrite anion via the pro-oxidative enzyme nitric oxide synthase (Gilad *et al.* 1997, Noda *et al.* 1999).

The granulosa cells and cumulus oocyte complexes (COCs) are the main sources of ovarian MTN (Reiter *et al.* 2007, Wang *et al.* 2021). Besides its antioxidative effects, MTN also regulates estradiol and progesterone production during the follicular and luteal phases, respectively (Chuffa, *et al.* 2011). MTN receptor 1 (MTNR1, also known as MT1) has been identified in oocytes and cumulus cells at GV and MII stages in mice, and supplementation of very low concentrations of MTN in murine oocytes reversed the effect of a meiosis-blocking agent, restoring meiosis *in-vitro* (Wang *et al.* 2021). In mammals, this occurs via its dedicated G- protein coupled MTN receptors ((MT1 (MTNR-1A) and MT2 (MTNR-1B)) (Reiter *et al.* 2007). MTNR-1A and acetyl serotonin O-methyltransferase (ASMT; melatonin producing enzyme) mRNA are expressed in both oocyte and cumulus cells in pigs and cattle (Kang *et al.* 2009, El-Raey *et al.* 2011). Indeed, treatment of COCs with luzindole (an inhibitor of MTN receptor) abolished the beneficial effects of MTN on oocyte maturation and development in pigs (Gao *et al.* 2019), cattle and juvenile goats (Soto-Heras *et al.* 2019).

In this study, we demonstrated the presence of MTN receptors in dog oocytes and the surrounding cumulus cells and analyzed the effect of different concentrations of MTN on oocyte nuclear maturation, ROS production and expression of ROS regulating genes after IVM in a low oxygen environment (low O_2_) at 38.5°C.

## Materials and methods

### Chemicals and reagents

All chemicals and reagents were purchased from Sigma Chemical Company (UK), unless otherwise stated.

### Collection of ovaries

**Figure 1:**
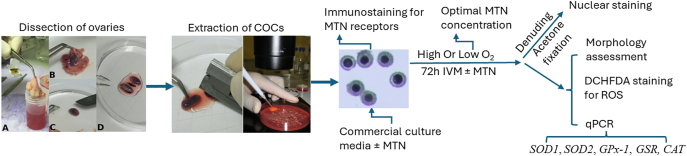
Steps in dissection of dog ovaries, extraction and culture of COCs and the analyses carried out in these studies. Oocytes were extracted through chopping of ovary cortex. Isolated COCs were used for immunostaining of MTN receptors, optimization of MTN concentration, comparing MTN effect in low or high O2 culture on oocyte morphology, nuclear maturation and qPCR analysis of ROS repairing enzymes and impact of MTN supplementation to commercial culture media.

**Figure 2:**
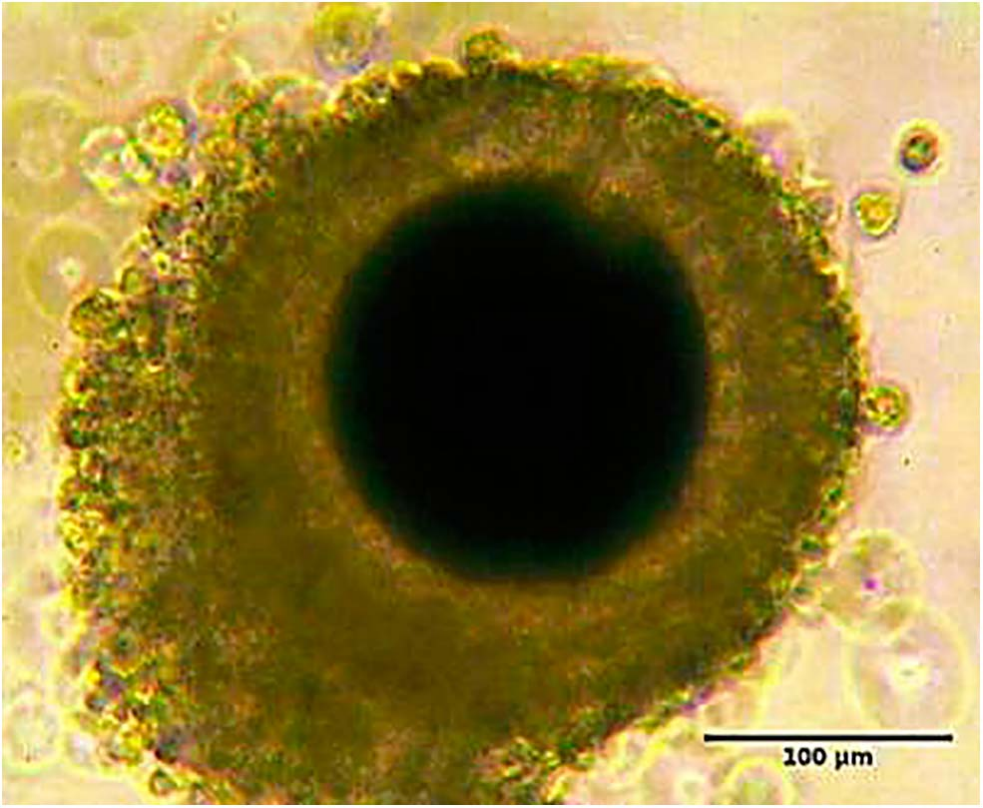
Photomicrograph of a freshly collected dog cumulus oocyte complex. The COC is characterized by a dense layer of cumulus cells surrounding the oocyte with dark cytoplasm.

This study was conducted after approval of the project by the Clinical Research Ethical Review Board of the Royal Veterinary College (URN 2019 1872-2) and with owner-informed consent. Collection of dog ovaries and preparation for COC culture were performed, as previously described (Salavati *et al.* 2012), in pet dogs undergoing routine ovariectomy (for reasons unrelated to our study). Sample collection was blind to the reproductive stage of the dog (*n* = 35), and her breed, age, weight and size. Ovaries were collected immediately after ovariectomy and placed in a 60 mL container (VWR international, USA) containing 30 mL (37°C) sterile phosphate buffered saline (PBS) and transferred to the laboratory within one hour (h). Before dissection of follicle and oocytes, the ovaries were washed in sterile 37°C PBS (1× PBS made in house from 20× concentrated PBS containing 2.74 M NaCl, 54 mM KCl, 160 mM Na_2_HPO_4_ and 29.2 mM NaH_2_PO_4_) and trimmed of the ovarian bursa and other debris using a sterile scalpel blade and washed in synthetic oviductal fluid (SOF) culture medium supplemented with HEPES (20 mM) and bovine serum albumin (BSA) (4 mg/mL). The process of oocyte extraction from dog ovaries and the analyses that were carried out in the present study is depicted in Fig. 1. The COCs were dissected after slicing the ovaries gently using a set of multiple blades (Salavati *et al.* 2013). COCs with oocyte diameter over 100 μm excluding the ZP, surrounded by at least three layers of cumulus cells with dense and homogenous ooplasm were selected for IVM and washed twice in the dissection medium before culture (Fig. 2).

### Immunocytochemistry for melatonin receptors

A group of freshly collected COCs from different animals were denuded partially (*n* = 60 in total) by passing through 200 μm flexi pipette to improve visibility of oocytes after staining. They were fixed in 4% paraformaldehyde in PBS for 20 min before three sequential washes each in 2 mL PBS and processing for immunocytochemistry, as previously described (Fouladi-Nashta *et al.* 2008). In brief, all steps of this experiment were performed at room temperature (RT; 22°C) unless stated. Nonspecific binding was blocked by incubation in 3% BSA (A8806) in PBS (without Ca^2+^ and Mg^2+^ (^–Ca–Mg^) (v/v)) for 2 h on a shaker. Subsequently, the COCs and oocytes were incubated in a humidified chamber at 4°C overnight in polyclonal rabbit anti-MTNR1A (bs-0027A) or anti-MTNR1B (bs-0963R) antibodies (BiOSS Woburn, USA) diluted in the blocking solution (1:50 v/v) alongside two negative controls: i) only blocking solution without primary antibody and ii) rabbit IgG in blocking solution (1:1,000 v/v). It is noteworthy that no peptide block controlled was used in this study both based on manufacturer’s recommendation and lack of any significant pBLAST similarity with other epitopes, refer to Supplementary file 1X (see section on Supplementary materials given at the end of the article). On the following day, they were washed briefly in PBS^–Ca–Mg^ before incubating for 1 h with secondary antibody (FITC-conjugated polyclonal goat anti-rabbit IgG (fab fragment, Jackson Immuno-Research)) (1:1,000 v/v). Afterward, these COCs and oocytes were washed three times in 0.2% Triton in PBS^–Ca–Mg^ (v/v) and then incubated in 0.2% Triton in PBS with 10 μg/mL Hoechst 33342 for 20 min in the dark on a shaker. They were placed on a glass slide beneath coverslips fixed with four paraffin/Vaseline drops (1/40; v/v) and mounted using anti-fading Vectashield mounting medium (Vector laboratories, USA) and visualized at 360–470 nm LED lamp under an Olympus BX60 fluorescence microscope.

### IVM of oocytes and optimization of melatonin concentration

The selected COCs (maximum of 25/well) were cultured for 72 h at 38.5°C in low O_2_ (5% CO_2_, 5% O_2_, 90% N_2_) or high O_2_ (5% CO_2_ in air), as previously described (Salavati *et al.* 2012), in four-well tissue culture dishes (NUNC, Thermo Fisher Scientific, USA). The base maturation medium was SOF medium plus amino acids (SOFaaci) (Fouladi-Nashta *et al.* 2007) supplemented with luteinizing hormone 5 μg/mL (Leutropin; Bioniche Animal Health, Canada), follicle stimulating hormone 5 μg/mL (Follitropin; Bioniche Animal Health, Canada), 17β-estradiol 1 μg/mL (E2), progesterone 1 μg/mL (P4), 50 μg/mL gentamicin and 6 mg/mL BSA.

To optimize MTN concentration, dog COCs (*n* = 295) were cultured in a low O_2_ incubator in the absence (control) or presence of increasing concentrations of 1 nM, 100 nM or 10 μM MTN supplemented to oocyte maturation medium. Oocyte nuclear maturation through meiosis was determined as described below.

### Denuding oocytes and assessment of nuclear maturation

Oocytes were mechanically denuded from the cumulus cells by gently pipetting up and down in a 10 μL Gilson pipette tip, followed by further denudation using a flexi pipette (135 μm luminal diameter) (CooperSurgical Company, USA). The denuded oocytes were placed in a drop of HEPES-SOF on Superfrost slides (VWR International, UK) and air-dried at RT for 20 min. Afterward, the oocytes were fixed in chilled (−20°C) 99% acetone (Merck Life Science, UK) for 10 min. Hoechst 33342 fluorescent DNA dye was prepared in PBS at 10 μg/mL for simultaneous staining and rehydration of slides for 5 min at RT. Oocytes were mounted using anti-fading Vectashield mounting medium beneath a coverslip and observed under the fluorescence microscope as explained earlier. After determining their nuclear stage in meiosis, they were categorized into seven groups from GV to MII and the number of oocytes at each stage was recorded. Degenerated oocytes with undetermined, absent or morphologically abnormal nuclear material were categorized as a separate group (‘Degen’) for all experiments. A panel of stained oocytes at different meiotic stages, shown by Salavati *et al.* (2012), was used as a recognition guide for all experiments.

### ROS staining using dichlorodihydrofluorescein diacetate (DCHFDA)

**Figure 3:**
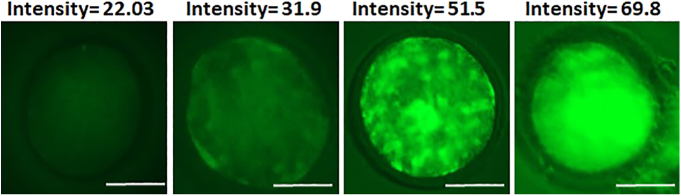
Representative images of oocytes with different intensity of ROS staining. Scale bar = 50 μm.

COCs cultured in high- and low-O_2_ groups were partially denuded after 72 h of culture and stained using DCHFDA. DCHFDA is hydrolyzed (via intracellular esterase) to 20,70-dichlorodihydrofluorescein (DCHF); the latter metabolite is then oxidized (via H_2_O_2_) to 20,70-dichlorofluorescein (DCF) which glows in green flourescence (Nasr-Esfahani *et al.* 1990, Wakefield *et al.* 2008). The COCs were washed twice in 0.04% polyvinyl pyrrolidone (PVP) in PBS and then incubated in 0.04% PVP-PBS buffer containing 10 mM DCHFDA for 30 min at 38.5°C in the dark. These COCs were washed twice in the same buffer and mounted on slides using anti-fading Vectashield mounting medium under a coverslip and were visualized by a 470 nm LED lamp olympus BX60 fluorescence microscope. DCF in these COCs underwent excitation at 470 nm wavelength, and the emission (at 522–530 nm) was captured by fluorescence microscopy. The partial denudation allowed visualization of both the oocyte and cumulus cells after staining. Images were captured from 25 COCs of high- and low-O_2_ groups in each repeat. The fluorescence intensity was quantified using the ImageJ software (particle analysis plug-in) (Abramoff *et al.* 2004), as presented in Fig. 3.

### Real time PCR and expression profile of ROS repair enzymes

Expression of genes encoding ROS repair enzymes superoxide dismutase (*SOD1* and *SOD2*), *GPX-1*, *GSR* and catalase (*CAT*) were analyzed in freshly collected COCs (control; 0 hour culture; 0 h) and COCs cultured in SOFaaci maturation medium in the absence or presence of the optimized MTN concentration of 100 nM for 72 h. Genomic sequences were obtained from NCBI and primers were designed using the Primer3 (Rozen & Skaletsky 2000) web-based software (Table 1). Overall, 300 COCs were used in this experiment. A proportion of the collected COCs were snap frozen immediately after dissection (uncultured control; 0 h, *n* = 25 from each batch, *n* = 100 over four repeats). The remainder were cultured (25 COCs per treatment/replicate, *n* = 200 in total) for 72 h, subsequently washed in 0.4% PVP, snap-frozen and maintained in liquid nitrogen and stored at until analysis. RNA was extracted using a QIAGEN RNeasy kit (QIAGEN, UK), according to the manufacturer’s instructions. RNA concentration was measured using a TECAN plate reader (TECAN, Switzerland) and adjusted by dilution to 50 ng in 8 μL volume before reverse transcription (RT) using the QIAGEN Sensiscript RT kit (total reaction volume 20 μL). Subsequently, PCR was conducted with 12.5 ng cDNA per reaction in duplicate and with four sets of repeats. Relative realtime qPCR was carried out using a previously described protocol (Salavati *et al.* 2012) and canine GAPDH as the house keeping gene, which had a stable expression among three groups (control (0 h) and the two treatments) in four replicates. (Levene’s test *P* = 0.086; one-way ANOVA (equal variances assumed; LSD post-HOC); *P* value = 0.227). The fold induction (expression) of target genes was analyzed using the Livak method (2^−ΔΔCT^) (Livak 2001).

**Table 1:** Sequence of the designed primers and accession numbers of the ROS repairing enzyme genes.

Canine genes	Accession number	Oligos (5′ → 3′)		Product size (bp)
		Forward	Reverse	
*SOD1*	NM_001003035.1	ACC​ATT​ACA​GGG​CTG​ACT​GAA​G	TGG​ACA​GAG​GAT​TAA​AGT​GAG​GA	115
*SOD2*	XM_533463.3	AGA​AGG​GTG​ACA​TTA​CAG​CTC​A	AAT​CAC​GTT​TGA​TGG​CTT​CC	153
*GPX1*	NM_001115119.1	GAC​ACC​ACT​GCG​CTA​ATG​AC	AGG​GAA​AGG​AGG​GTT​GCC​TA	215
*GSR*	XM_532813.3	CTA​CGT​GAG​CCG​CCT​AAA​TAC	CTG​TGG​CAA​TCA​GGA​TGT​GAG	155
*CAT*	NM_001002984.1	CCC​ATT​GCA​GTT​CGA​TTC​TC	CTA​TGG​ATA​AAG​GAC​GGA​AAC​A	179
*GAPDH*	NM_001003142.1	GTG​ATG​CTG​GTG​CTG​AGT​ATG​T	ATG​GAT​GAC​TTT​GGC​TAG​AGG​A	233

ROS, reactive oxygen species.

### Melatonin supplementation to commercial culture media used in human assisted reproduction

The efficacy of two commercial IVM media used for human oocytes (G-IVF PLUS culture medium (contains triple antioxidants; 10 μmol/L acetyl-L-carnitine, 10 μmol/L N-acetyl-l-cysteine and 5 μmol/L alpha-lipoic acid) (Vitrolife, Sweden)) and ORIGIO-V1 culture medium (CooperSurgical, UK) were compared to SOFaaci, with 670 COCs in the presence or absence of 100 nM MTN. Morphology of the COCs after 72 h of cultures and nuclear maturation of the oocytes were assessed after denuding fixing in chilled acetone, followed by Hoechst 33342 staining as detailed earlier.

### Statistical analysis

All experiments were repeated at least three times. The proportional average of oocytes at different stages of meiotic resumption was calculated at the end of the culture period in comparison with the total number of cultured oocytes. Statistical analysis was conducted using the PAWS statistics 24 and Statistical Package for Social Sciences (SPSS Inc., USA) using binary and ordinal logistic regressions via generalized linear models. Repeat (dogs) were considered as random variables in the analysis to account for repeated measurements within each dog’s ovaries. Differences among treatment groups were considered significant if *P* values were <0.05. Data are presented as the mean ± SEM.

## Results

### Melatonin receptor (MTNR-A1 and -1B) localization

**Figure 4:**
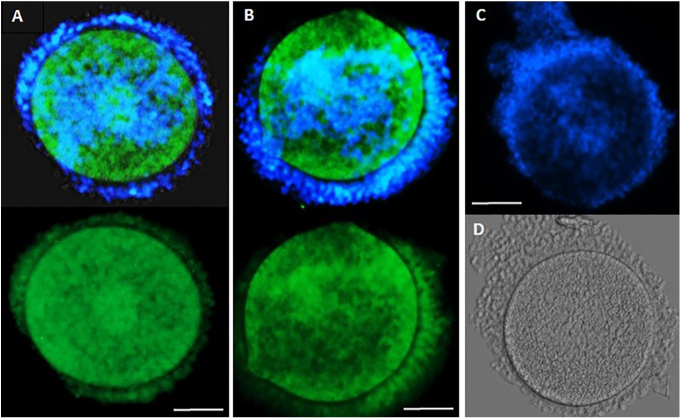
Immunolocalization of MTNR-1A and -1B. Fluorescent microscope images of COCs after immunocytochemistry for MTNR-1A and -1B. MTNR-1A showed stronger FITC staining (green) in the ooplasm than the MTNR-1B. However, the MTNR-1B exhibited stronger FITC staining in the cumulus cells than the MTNR-1A. Image C = negative control, image D: light microscopy image of the COC in image C. Scale bar = 40 μm.

In total, 50 partially denuded COCs were stained to visualize the localization of MTNR-1A and -1B (25 COCs for each receptor). MTNR-1A was highly and consistently expressed in the oocytes (Fig. 4A) and with lower intensity in the cumulus cells. The very first layers of cumulus cells had also weak signal of the MTNR-1A (lower image Fig. 4A). However, MTNR-1B was highly and consistently expressed at similar level, both in the oocytes and cumulus cells (Fig. 4B).

### Effects of MTN concentration on nuclear maturation of dog oocytes

**Figure 5:**
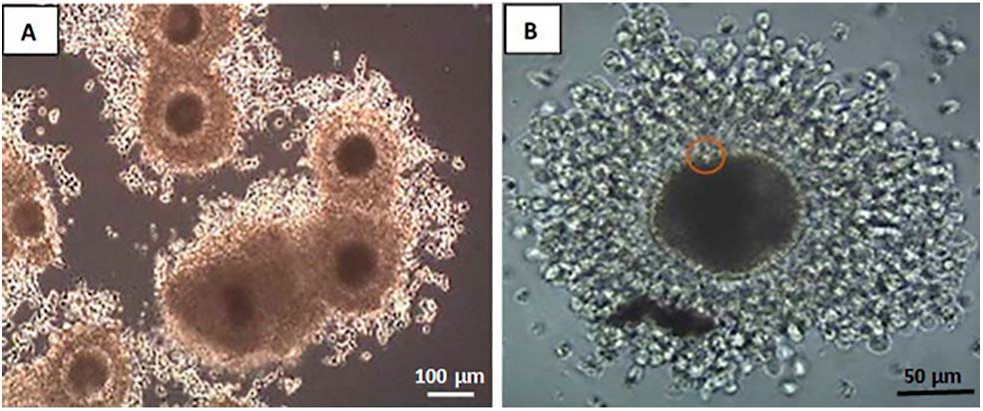
Dog COCs with expanded cumulus cells after IVM (A) and sunburst cumulus cells and mature oocytes with an extruded polar body (red circle in the picture on the right) (B).

As detailed in Table 2, a total number of 295 COCs were cultured in a low O_2_ incubator in the absence (control) or presence of increasing concentrations of MTN: 1 nM, 100 nM and 10 μM. Melatonin at 100 nM was optimal in terms of nuclear maturation of dog oocytes after 72 h culture with the lowest percentage of oocytes remaining at GV stage (6.7% ± 4.2), highest MII maturation rate (32.3% ± 6.4), minimum degeneration (20.5% ± 3.2) and maximal meiotic resumption (56.2% ± 8.6) among COCs cultured in all other conditions (*P* < 0.05) (Table 2). Oocytes cultured in 100 nM MTN exhibited a significant difference with those cultured in control condition (*P* < 0.05). These oocytes showed expansion of cumulus cells (Fig. 5A) and extruded polar bodies (Fig. 5B). The MII maturation rate of oocytes was also significantly higher in all MTN-treated COCs as compared to the control group (*P* < 0.05). The positive effect of MTN in enhancing meiotic resumption was also evident at 10 μM and 1 nM (both *P* < 0.05); however, in these latter two concentrations of MTN, the oocyte degeneration rate remained like that of the control group. Overall, based on these results, 100 nM MTN concentration was selected to be used in all subsequent experiments.

**Table 2:** Effect of melatonin concentration on nuclear maturation/degeneration of dog IVM oocytes cultured in low O_2_ concentrations. The oocytes were cultured in SOFaaci medium supplemented with 100 nM melatonin for 72 h at 38.5°C in 20% or 5% O_2_ incubator. Results are presented as the percentage of oocytes (mean ± SEM) in different meiotic stages at 72 h in hypoxic condition.

	GV	GVBD	MI	AI	TI	MII	Degen	MI–MII^†^	*n*
Control	15.5 ± 4.2	27.6 ± 8.4	8.5 ± 4.8	0.0 ± 0.0	6.6 ± 4.6	11.8 ± 2.6	29.9 ± 7.4	27.0 ± 3.2	70
1 nM MTN	10.4 ± 5.5	20.1 ± 2.5	15.7 ± 8.8	0.0 ± 0.0	4.6 ± 2.3	21.1 ± 6.3*	28.0 ± 6.1	41.4 ± 12.8	68
100 nM MTN	6.7 ± 4.2*	16.6 ± 1.3	11.9 ± 1.2	3.2 ± 1.7*	8.7 ± 2.8	32.3 ± 6.4*	20.5 ± 3.2*	56.2 ± 8.6*	80
10 μM MTN	9.1 ± 4.9*	23.3 ± 3.4	8.1 ± 4.4	0.7 ± 0.7	4.0 ± 2.0	26.0 ± 4.8*	28.7 ± 6.1	38.8 ± 6.4*	77
Total									295

MTN, melatonin; Degen, degenerated; GV, germinal vesicle; GVBD, germinal vesicle breakdown; MI, metaphase 1; AI, anaphase 1; TI, telophase 1; MII, metaphase 2; IVM, *in vitro* maturation.

* *P* < 0.05 within the column compared to control.

^†^ Indicates meiotic resumption.

### Effects of O_2_ concentration and melatonin on oocyte maturation and oocyte morphology

**Figure 6:**
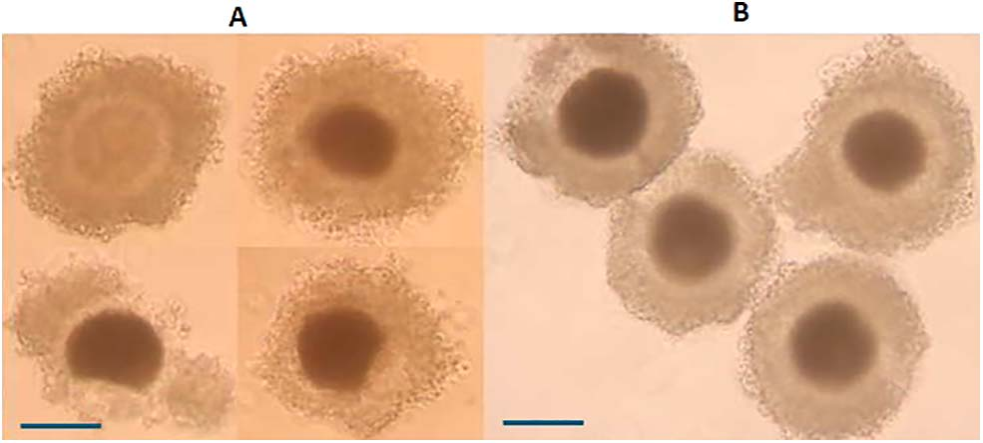
Images of dog COCs cultured 72 h in SOFaaci medium at high O_2_ (A) and low O_2_ (B) incubator. The COCs cultured in high O_2_ appeared with many deformed oocytes and dispersing cumulus cells. Scale bar = 100 μm.

A total of 152 oocytes were cultured in this experiment. COCs cultured in low O_2_ (*n* = 80) exhibited the lowest percentage of oocytes at GV stage ((6.7% ± 4.2 vs 19.8% ± 3), *P* < 0.01), highest MII maturation rate ((32.3% ± 6.4 vs 15.81% ± 8.1), *P* < 0.05), lower degeneration ((20.5% ± 3.2 vs 45.2% ± 5.15), *P* < 0.01) and higher meiotic resumption ((GVBD-MII; 56.2% ± 8.6 vs 19% ± 3), *P* < 0.01) in SOFaaci maturation medium supplemented with 100 nM MTN (Table 3). Many oocytes within the COCs cultured in high O_2_ looked deformed and showed signs of degeneration after 72 h IVM (Fig. 6A) when compared to the low O_2_ group (Fig. 6B) (*n* = 70).

**Table 3:** Effect of oxygen concentrations and melatonin on nuclear maturation of dog oocytes after 72 h. Table shows distribution of oocytes in different stages of meiotic division. Results are presented as the percentage of oocytes (mean ± SEM) in different meiotic stages after 72 h IVM in SOF with 100 nM melatonin in low oxygen vs high oxygen conditions.

	High O_2_	Low O_2_
GV	19.8 ± 3	6.7 ± 4.2^†^
GVBD	20.1 ± 2.5	16.6 ± 1.3
MI	3 ± 2.8*	11.9 ± 1.2
AI	0.0 ± 0.0	3.2 ± 1.7*
TI	0.5 ± 2.2*	8.7 ± 2.8
MII	15.8 ± 8.1	32.3 ± 6.4*
Degen	45.2 ± 5.15	20.5 ± 3.2^†^
MI–MII^‡^	19.4 ± 3	56.2 ± 8.6^†^
*n*	72	80

GV, germinal vesicle; GVBD, germinal vesicle breakdown; MI, metaphase 1; AI, anaphase 1; TI, telophase 1; MII, metaphase 2; *n*, total number of oocytes in each treatment group; MTN, melatonin, Degen, degenerated; IVM, *in vitro* maturation; SOF, synthetic oviductal fluid.

* *P* < 0.05.

^†^ *P* < 0.01.

^‡^ Indicates meiotic resumption of oocytes (total number of oocytes between MI and MII).

### Effect of melatonin on ROS production

**Figure 7:**
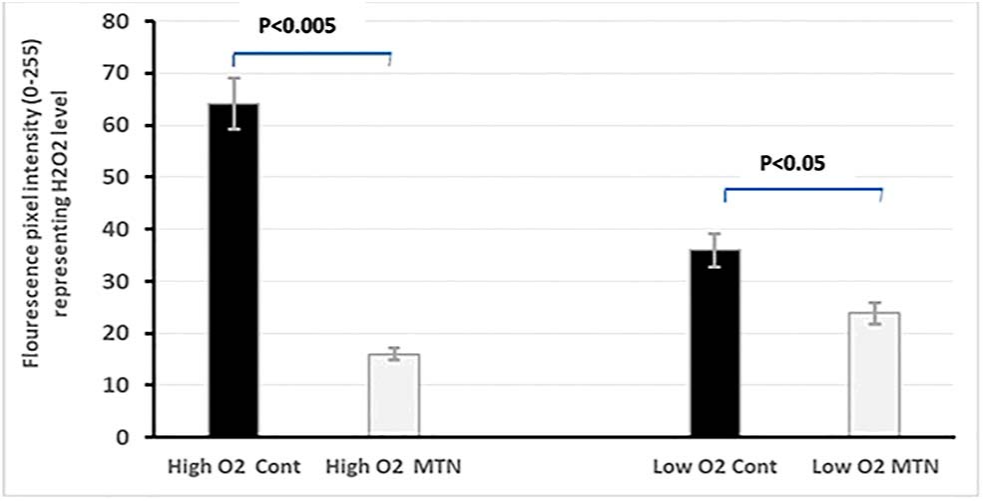
Effect of 100 nM melatonin on H_2_O_2_ production in dog IVM oocytes cultured 72 h in high O_2_ (5% O_2_, 5% CO_2_) concentration vs low O_2_ (5% O_2_, 5% CO_2_ and 90% N_2_).

The effect of MTN on production of ROS was analyzed using DCHFDA staining in partially denuded COCs (*n* = 50). Densitometry using the ImageJ software showed that the overall intensity of fluorescence was lower in oocytes treated with 100 nM MTN ((*P* < 0.05) Fig. 7). This effect was more pronounced in oocytes cultured in high O_2_ when compared to the low O_2_ group (*P* < 0.005).

### Effect of melatonin supplementation to commercial culture media on oocyte nuclear maturation

The efficacy of two commercial IVM media used for human oocytes (G-IVF PLUS culture medium (contains triple antioxidants; 10 μmol/L acetyl-L-carnitine, 10 μmol/L N-acetyl-l-cysteine and 5 μmol/L alpha-lipoic acid) (Vitrolife, Sweden))) and ORIGIO-V1 culture medium (CooperSurgical) were compared to SOFaaci, with 670 COCs in the presence or absence of 100 nM MTN. Supplementation of MTN to G-IVF PLUS and SOF medium increased oocyte development to MII stage (*P* < 0.05). Supplementation of MTN to SOF medium reduced the number of degenerated oocytes (*P *< 0.05). But it had no effect on oocyte degeneration rate when supplemented to G-IVF PLUS medium. Most of the oocytes cultured without MTN in ORIGIO-V1 demonstrated degeneration (*P* < 0.05) compared to the other two culture conditions. Importantly, none of these oocytes were found at GV or GVBD stages. MTN supplementation in ORIGIO-V1 significantly increased meiotic resumption and reduced oocyte degeneration (*P* < 0.05) but not the number of oocytes that developed to the MII stage (Table 4).

**Table 4:** Effect of melatonin supplementation to commercially available human oocyte maturation medium on nuclear maturation of dog IVM oocytes. Melatonin at 100 nM concentration was added to dog COCs cultured for 72 h in low O_2_ incubator in SOFaaci medium (control) or G-IVF PLUS or ORIGIO V1 media. Results are presented as the percentage of oocytes (mean ± SEM) in different meiotic stages at 72 h in hypoxic condition in commercial oocyte maturation medium ±100 nM MTN. Statistically significant values are presented in bold.

	GV	GVBD	MI	AI	TI	MII	Degen	MI–MII^†^	*n*
SOFMM	15.5 ± 4.2	27.6 ± 8.4	8.6 ± 4.8	0 ± 0	6.6 ± 4.6	11.8 ± 2.6	29.9 ± 7.4	54.2 ± 2.8	70
SOFMM +100 nM MTN	6.7 ± 1.2*	16.6 ± 1.3	12 ± 1.2	3.2 ± 1.7*	8.7 ± 2.8	32.3 ± 6.4*	20.5 ± 3.2*	72.8 ± 0.4	80
G-IVF PLUSE	23 ± 9	5 ± 1	16 ± 3	7 ± 4	4 ± 2	25 ± 3*	20 ± 4	57 ± 0.3	110
G-IVF PLUSE +100 Nm MTN	22 ± 8.8	3 ± 0	10.4 ± 6.74	10.8 ± 5.8	0 ± 0	36 ± 7.5*	17.8 ± 14.1	60.2 ± 1	153
ORIGIO V1	0 ± 0	0 ± 0	1.6 ± 0.5	1.5 ± 0.3	0 ± 0	10.6 ± 0.7	86.3 ± 10.6*	13.7 ± 2.3	113
ORIGIO V1 & 100 nM MTN	30 ± 13	8.9 ± 1	7.4 ± 2	7.4 ± 1	12.5 ± 3	8.8 ± 7	25 ± 15	45 ± 0.4*	144
Total *n*									670

SOFMM. SOFaaci medium; GV, germinal vesicle; GVBD, germinal vesicle breakdown; MI, metaphase 1; AI, anaphase 1; TI, telophase 1; MII, metaphase 2; *n*, total number of oocytes in each treatment group; MTN, melatonin, Degen, degenerated; IVM, *in vitro* maturation; COCs, cumulus oocyte complexes.

* *P* < 0.05 within the column compared to control.

^†^ Indicated meiotic resumption.

### Effect of melatonin on genes regulating ROS in COCs

**Figure 8:**
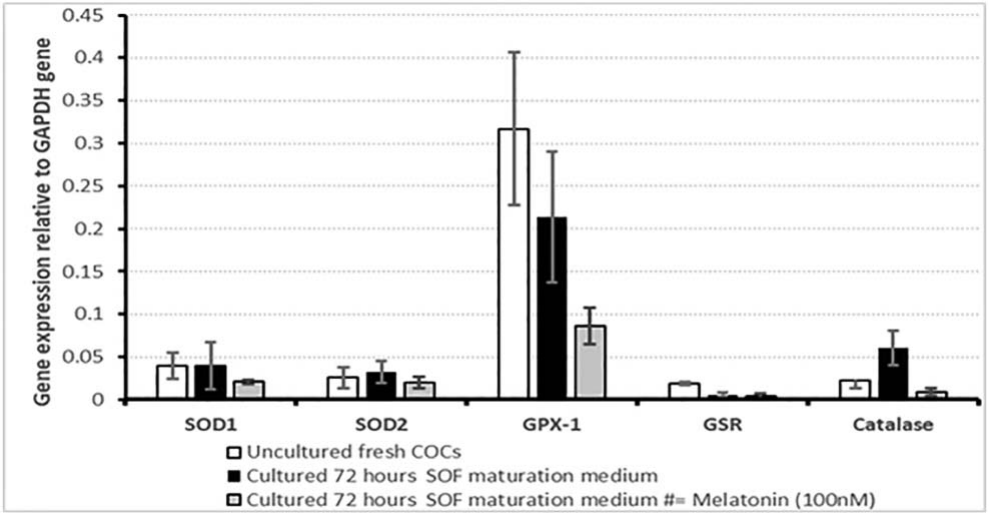
Effect of melatonin on mRNA expression profile of ROS repairing enzymes in canine COCs after 72 h of IVM compared to 0 h and normalized to expression of *GAPDH*. This figure shows the fold induction of ROS repairing enzymes; *GPX-1* (*P* < 0.05) and catalase (*P* < 0.05) in the absence of melatonin in dog COCs during the 72 h IVM. *SOD1*, cytosolic superoxide dismutase; *SOD2*, mitochondrial superoxide dismutase; *GSR*, glutathione reductase; *GPX-1*, glutathione peroxidase; *CAT*, catalase.

The impact of MTN supplementation on the expression of genes related to ROS repairing enzymes (*GPX-1*, *SOD1*, *SOD2*, *GSR* and *CAT*) was analyzed by qRT-PCR. Freshly collected COCs (control = 0 h) immediately after dissection and after 72 h culture were snap-frozen (25 COCs per group/four repeats). *GPX-1* was highly expressed in the control (0 h) COCs and in the COCs cultured in SOF without MTN. MTN supplementation reduced *GPX-1* (*P* < 0.05) and *CAT (P* < 0.05) expression in the COCs cultured in low O_2,_ although we found no difference in expression of the other genes (Fig. 8).

## Discussion

Unlike with other domestic animals, IVM of dog oocytes results in very low nuclear maturation and high degeneration rates; consequently, managing the degeneration is essential. The high-fat content of dog oocytes predisposes them to OS and to production of high levels of ROS, which have detrimental effects on oocyte health and developmental competence (Rodriguez *et al.* 2007, Songsasen & Wildt 2007, Salavati *et al.* 2012). MTN, as a highly lipophilic hormone, acts through its G-protein coupled receptors to function as a strong antioxidant to protect cells against OS (Dubocovich *et al.* 2003); it also regulates signaling mechanisms and transcription of genes critical for cellular functions. Data presented here show that both MTN receptors MTNR-1A and -1B are present in the oocyte and cumulus cells of canine COCs. Based on visual assessment, MTNR-1A staining intensity was higher in oocytes than in the cumulus cells, although MTNR-1B staining was similar in both cell types. The beneficial effect of MTN on dog oocytes could be mediated through either or both of these receptors.

In this study, MTN supplementation increased the proportion of oocytes that matured to the MII stage at three different concentrations when compared to oocytes cultured without MTN. This result corroborates previous studies in goats (Song *et al.* 2016) and in cattle (El-Raey *et al.* 2011). Importantly, oocytes from these species contain less lipids, require shorter duration of culture (24–25 h) and the oocytes are retrieved mainly through aspiration of ovarian follicles, which is not an invasive/traumatic procedure. In contrast, canine oocytes are cultured under sub-optimal maturation conditions, which exacerbate the interplay between lipid content and OS. This issue further underpins the requirements for additional support either through chemical supplementation, e.g. MTN, or different incubation conditions compared to other maturation models.

Although the beneficial effect of MTN during oocytes maturation has been attributed to its stimulatory effect on activity of intracellular antioxidant enzymes in mitochondria such as GSH, GR and glutathione peroxidase (Escames *et al.* 2006, Rodriguez *et al.* 2007; Remião *et al.* 2016), increasing adenosine triphosphate (ATP) formation as the source of energy for the oocytes (Remião *et al.* 2016, Song *et al.* 2016, Yu *et al.* 2017) may also be involved. It is known that mammalian COCs produce ATP mainly through glycolysis and the pentose phosphate pathway to provide energy required during oocyte maturation (Downs 1995, Sutton-McDowall *et al.* 2010). Specifically in dogs, it was shown that glycolytic activity during maturation *in vitro* increases as the oocyte progresses from GV to MII stage (Songsasen *et al.* 2012). The role of MTN supplementation on stimulating energy production during IVM requires detailed investigation.

Previous reports showed that MTN addition to culture media improves cellular mitochondrial distribution and increased expression of mitogen-activated protein kinases (MAPKs) and maturation promoting factor (MPF) gene components in porcine IVM oocytes (Zhao *et al.* 2018). In mice IVM, MAPK and MPF pathway activation is accompanied by elevated levels of cyclic adenosine monophosphate (cAMP) concentration in cumulus cells, reduced delivery of cyclic guanosine monophosphate (cGMP) to the oocytes and increased transition of oocytes from GV stage to GVBD (Guraya 1965). These lead to resumption of meiosis (Songsasen & Wildt 2007, Rodrigues & Rodrigues 2010) accompanied by downregulation of natriuretic peptide receptor-2 (NPR2) and blocking the gap junctions between the cumulus cells and oocytes (Sun *et al.* 2016). MAP kinases are involved in the regulation of microtubule dynamics, especially the maintenance of metaphase organization (Songsasen *et al.* 2017), and have a role in the initiation of translation. Other studies have shown that MAPK activation results in higher oocyte nuclear maturation rates in COCs derived from small follicles (Zhao *et al.* 2020). In this context, although we have not analyzed MAPKs or cAMP in the present study, a higher level of ERK1/2 phosphorylation in MTN-treated COCs could mediate the beneficial MTN effect directly or indirectly (through cAMP or by supporting oocyte health).

Glutathione (GSH) is the main non-enzymatic cellular defense mechanism against ROS and other free radicals (Menezo *et al.* 2010). GSH becomes a substrate of glutathione peroxidase (GPX) in alliance with catalase (CAT), which degrades H_2_O_2_ to water and oxygen (Whitaker & Knight 2008). H_2_O_2_ itself is the resulting product of neutralization of ROS by SOD present in the cytoplasm (SOD1) and mitochondria (SOD2) (Guerin *et al.* 2001). To recover GSH, oocytes use another enzyme, GR, to reduce the GS-SG (glutathione disulfide oxidized form of GSH) back to GSH. Because of lower levels of GSH within *in vitro* matured canine oocytes (<8 pMol/oocyte) in comparison with *in vivo* (19.2 pMol/oocyte) (Kim *et al.* 2007), high levels of H_2_O_2_ and ROS within COCs during IVM can severely impair maturation and increase degeneration rates (Whitaker & Knight 2008). Therefore, the H_2_O_2_ level in oocytes is a good indication of OS.

In dogs, the oocytes mature after ovulation within the microenvironment of the oviduct. There is no report about oxygen tension within dog’s oviduct. Overall, the oxygen tension within the oviduct has been reported to vary between 4 and 10% across species (Huo *et al.* 2024), with specific values of 5.3–6% in the hamster (Fischer & Bavister 1993), 7.0–7.9 in rabbits (Mastroianni & Jones 1965) and 7.6% in pigs (Garcia-Martinez *et al.* 2018), although there are no data for the dog. To assess the impact of MTN on oocyte heath, we quantified the abundance of their ROS production after IVM by performing ROS staining using DCHFDA on two groups of COCs cultured in SOF medium, with and without 100 nM MTN in low O_2_ and high O_2_ conditions. The oocytes cultured in high O_2_ environment without MTN showed higher intensity of DCHFDA staining compared with the COCs cultured in low O_2_ environment, revealing higher hydrogen peroxide production in cells in a high O_2_ environment. However, MTN (100 nM) supplementation not only reduced the level of OS in the IVM oocytes cultured in high O_2_, but it also had a similar effect on the oocytes cultured in low O_2_. On the other hand, the maturation rate of the COCs cultured in low O_2_ condition was also over two-fold higher than the oocytes cultured in high O_2_ (Table 3). Importantly, a higher proportion of the oocytes cultured in high O_2_ showed morphological defects, with a degenerative appearance when compared to those in low O_2_ (Fig. 4). This data replicates our previous results, where high O_2_ increased the degeneration rate of canine oocytes and their ROS production (Salavati *et al.* 2012).

To investigate whether MTN could reduce levels of OS in IVM oocytes, we examined the expression of OS-associated genes. From the five genes studied, MTN supplementation downregulated mRNA expression of *GPX-1* and catalase. High *GPX-1* expression in uncultured and cultured COCs without MTN might be evidence of high organic compound peroxidation and the generation of H_2_O_2_ free radicals (Pei *et al.* 2023). However, catalase gene expression was significantly higher only in the COCs cultured in SOF without MTN. This could reflect higher H_2_O_2_ generation during culture, which was reduced in MTN-supplemented oocytes. Overall, the antioxidant properties of MTN in dog oocytes seems to be mediated via activating antioxidant response elements (ARE system), inducing gene expression and production of antioxidant enzymes, i.e., *GPX-1* (Chrustek 2021), and catalase to create a balance between the generation of ROS, such as O_2_ and H_2_O_2_ (respectively) in oocytes.

We also examined the effect on canine oocyte nuclear maturation of adding MTN to two commercially available IVM media used for human oocytes. Oocytes cultured in G-IVF PLUS with and without supplementation of 100 nM MTN had slightly higher maturation rates and lower degeneration rates than oocytes cultured in SOFaaci culture medium. The positive effect of G-IVF medium could be due to its antioxidant content (acetyl-L-carnitine, alpha lipoic acid and N-acetyl-L-cysteine). We have previously reported the beneficial antioxidant effect of alpha-lipoic acid on protecting *in vitro* cultured ovine oocytes through increasing the thiol content in oocytes (Moghimi *et al.* 2021). N-acetyl-cysteine (NAC) is a cysteine precursor that suppresses oxidative damage by increasing the levels of glutathione (Hu *et al.* 2020), while MTN increases the expression of antioxidant enzymes and minimizes oocyte OS. L-carnitine stabilizes mitochondrial membranes and protects DNA from ROS-induced damage (Ismail *et al.* 2014). Overall, it seems that MTN supplementation to G-IVF has additive effects, which further improved maturation rates and reduced canine oocyte degeneration.

In conclusion, MTN at 100 nM in dog oocyte IVM improved oocyte heath and nuclear maturation. MTN supplementation benefits oocyte development even in a low oxygen environment and in the presence of other antioxidants. Further work is needed to understand the molecular mechanism of the impact of MTN on canine oocyte maturation and for assessing the developmental competence of these oocytes after fertilization.

## Supplementary materials



## Declaration of interest

The authors declare that there is no conflict of interest that could be perceived as prejudicing the impartiality of the work reported.

## Funding

Funding for this project was provided by Duchenne UK. The authors would like to acknowledge all the veterinary clinics and dog owners who contributed to this project by allowing access to material that would otherwise have been discarded.

## Author contribution statement

All authors contributed to the concept development and design of the experiments presented in this manuscript. Fataneh Ghafari and Mazdak Salavati carried out the experimental works, collected data, analyzed and interpreted data and prepared the draft of the manuscript. Ali Fouladi-Nashta and Richard Piercy were the project leaders, helped with sample collection and contributed to data analysis, critical revision improving and approving the manuscript.
