# Supplementary material for: Beneficial effects of melatonin on canine oocyte nuclear maturation via reduction of oxidative stress
Source: Reproduction. 2025 Mar 14;169(4):e240388. doi: 10.1530/REP-24-0388 (PMC11915559; doi:10.1530/REP-24-0388)
Supplement: Supplementary file 1 [file supplementary_materials.pdf]

Hit	DB	Accession	Description	Organism	Length	Score(Bits)	Identities(%)	Positives(%)	E()
1	SP	P49286	Melatonin receptor type 1B OS=Homo sapiens OX=9606 GN=MTNR1B PE=1 SV=1	Homo sapiens	362	1908	100	100	0
2	TR	A0A2R9ADD5	Melatonin receptor 1B OS=Pan paniscus OX=9597 GN=MTNR1B PE=3 SV=1	Pan paniscus	362	1894	98.9	99.4	0
3	TR	H2R515	Melatonin receptor 1B OS=Pan troglodytes OX=9598 GN=MTNR1B PE=3 SV=2	Pan troglodytes	362	1894	98.9	99.4	0
4	TR	G3RDH2	Melatonin receptor 1B OS=Gorilla gorilla gorilla OX=9595 GN=MTNR1B PE=3 SV=2	Gorilla gorilla gorilla	362	1870	98.1	98.6	0
5	TR	A0A2K6MKK9	Melatonin receptor 1B OS=Rhinopithecus bieti OX=61621 GN=MTNR1B PE=3 SV=1	Rhinopithecus bieti	362	1865	96.7	98.6	0
6	TR	A0A2K6NIS9	Melatonin receptor 1B OS=Rhinopithecus roxellana OX=61622 GN=MTNR1B PE=3 SV=1	Rhinopithecus roxellana	362	1865	96.7	98.6	0
7	TR	A0A096NB84	Melatonin receptor 1B OS=Papio anubis OX=9555 GN=MTNR1B PE=3 SV=1	Papio anubis	362	1860	96.7	98.3	0
8	TR	A0A2K5IJB8	G-protein coupled receptors family 1 profile domain-containing protein OS=Colobus angolensis palliatus OX=336983 PE=3 SV=1	Colobus angolensis palliatus	362	1860	96.4	98.3	0
9	TR	A0A8D2JZ74	Melatonin receptor 1B OS=Theropithecus gelada OX=9565 GN=MTNR1B PE=3 SV=1	Theropithecus gelada	362	1857	96.4	98.1	0
10	TR	A0A0D9S140	Melatonin receptor 1B OS=Chlorocebus sabaeus OX=60711 GN=MTNR1B PE=3 SV=1	Chlorocebus sabaeus	362	1854	96.1	98.1	0
11	TR	A0A2K5ZTJ9	Melatonin receptor 1B OS=Mandrillus leucophaeus OX=9568 GN=MTNR1B PE=3 SV=1	Mandrillus leucophaeus	362	1852	96.4	98.1	0
12	TR	A0A8C9IYX7	Melatonin receptor 1B OS=Piliocolobus tephrosceles OX=591936 GN=MTNR1B PE=3 SV=1	Piliocolobus tephrosceles	362	1849	96.4	98.1	0
13	TR	A0A2K5MO08	Melatonin receptor 1B OS=Cercocebus atys OX=9531 GN=MTNR1B PE=3 SV=1	Cercocebus atys	362	1847	96.1	98.1	0
14	TR	A0A2K6BUA8	Melatonin receptor 1B OS=Macaca nemestrina OX=9545 GN=MTNR1B PE=3 SV=1	Macaca nemestrina	362	1841	95.6	97.5	0
15	TR	G7PNG2	Melatonin receptor 1B OS=Macaca fascicularis OX=9541 GN=MTNR1B PE=3 SV=1	Macaca fascicularis	362	1841	95.6	97.5	0
16	TR	H2NEY5	Melatonin receptor 1B OS=Pongo abelii OX=9601 GN=MTNR1B PE=3 SV=2	Pongo abelii	361	1821	96.1	97.5	0
17	TR	A0AA41N2S2	Melatonin receptor type 1B OS=Sciurus carolinensis OX=30640 GN=SUZIE_167530 PE=3 SV=1	Sciurus carolinensis	364	1642	85.6	90	0
18	TR	F6YWF5	Melatonin receptor 1B OS=Equus caballus OX=9796 GN=MTNR1B PE=3 SV=2	Equus caballus	380	1637	84.6	90.9	0
19	TR	H0XTE5	Melatonin receptor 1B OS=Otolemur garnettii OX=30611 GN=MTNR1B PE=3 SV=1	Otolemur garnettii	364	1636	84.8	90.1	0
20	TR	A0A8C9DSG3	Melatonin receptor 1B OS=Prolemur simus OX=1328070 GN=MTNR1B PE=3 SV=1	Prolemur simus	363	1628	85.8	90.6	0
21	TR	I3MCE3	Melatonin receptor 1B OS=Ictidomys tridecemlineatus OX=43179 GN=MTNR1B PE=3 SV=1	Ictidomys tridecemlineatus	364	1624	84.3	90.4	0
22	TR	A0A8D2JSB8	Melatonin receptor 1B OS=Sciurus vulgaris OX=55149 GN=MTNR1B PE=3 SV=1	Sciurus vulgaris	364	1623	84.8	89.5	0
23	TR	A0A7J8H3K2	Melatonin receptor 1B OS=Rousettus aegyptiacus OX=9407 GN=HJG63_013695 PE=3 SV=1	Rousettus aegyptiacus	364	1614	84.1	90.4	0
24	TR	A0A8D2KBH0	Melatonin receptor 1B OS=Urocitellus parryii OX=9999 GN=MTNR1B PE=3 SV=1	Urocitellus parryii	362	1613	84.5	89.8	0
25	TR	L9JDP5	Melatonin receptor type 1B OS=Tupaia chinensis OX=246437 GN=TREES_T100018802 PE=3 SV=1	Tupaia chinensis	364	1609	84.3	89.8	0
26	TR	A0A8C5YYG2	Melatonin receptor 1B OS=Marmota marmota marmota OX=9994 GN=MTNR1B PE=3 SV=1	Marmota marmota marmota	362	1602	83.4	89.2	0
27	TR	A0A8C4M5Z9	Melatonin receptor 1B OS=Equus asinus OX=9793 GN=MTNR1B PE=3 SV=2	Equus asinus	380	1598	83.2	89.6	0
28	TR	A0A8C5Y9X6	Melatonin receptor 1B OS=Microcebus murinus OX=30608 GN=MTNR1B PE=3 SV=1	Microcebus murinus	364	1595	81.9	89	0
29	TR	A0A2K6EQL2	Melatonin receptor 1B OS=Propithecus coquereli OX=379532 GN=MTNR1B PE=3 SV=1	Propithecus coquereli	362	1589	83.6	88.3	0
30	TR	A0AA40LKE7	G-protein coupled receptors family 1 profile domain-containing protein OS=Eptesicus nilssonii OX=59451 GN=QTO34_004029 PE=3 SV=1	Eptesicus nilssonii	365	1585	81.9	89	0
31	TR	A0A6P3QC46	Melatonin receptor type 1B OS=Pteropus vampyrus OX=132908 GN=MTNR1B PE=3 SV=1	Pteropus vampyrus	355	1580	86.5	92.7	0
32	TR	A0A8C8XDC1	Melatonin receptor 1B OS=Panthera leo OX=9689 GN=MTNR1B PE=3 SV=1	Panthera leo	364	1578	82.7	89	0
33	TR	A0A8C9KEB3	Melatonin receptor 1B OS=Panthera tigris altaica OX=74533 GN=MTNR1B PE=3 SV=1	Panthera tigris altaica	364	1577	82.4	89	0
34	TR	A0A8B6YEJ2	Melatonin receptor type 1B OS=Camelus ferus OX=419612 GN=MTNR1B PE=3 SV=1	Camelus ferus	364	1576	81.7	89.5	0
35	TR	G1PIN7	Melatonin receptor 1B OS=Myotis lucifugus OX=59463 GN=MTNR1B PE=3 SV=1	Myotis lucifugus	364	1574	82.5	89	0
36	TR	A0A5N4DP71	Melatonin receptor type 1B OS=Camelus dromedarius OX=9838 GN=Cadr_000015407 PE=3 SV=1	Camelus dromedarius	364	1574	81.7	89.5	0
37	TR	L5L0B6	Melatonin receptor type 1B OS=Pteropus alecto OX=9402 GN=PAL_GLEAN10013775 PE=3 SV=1	Pteropus alecto	355	1572	86.2	92.4	0
38	TR	A0A6J0A6I9	Melatonin receptor type 1B OS=Acinonyx jubatus OX=32536 GN=MTNR1B PE=3 SV=1	Acinonyx jubatus	364	1572	82.4	88.7	0
39	TR	A0A667HN93	Melatonin receptor 1B OS=Lynx canadensis OX=61383 PE=3 SV=1	Lynx canadensis	364	1571	82.1	89	0
40	TR	F1STM6	Melatonin receptor 1B OS=Sus scrofa OX=9823 GN=MTNR1B PE=3 SV=3	Sus scrofa	364	1570	80.1	89.5	0
41	TR	M3VZW6	Melatonin receptor 1B OS=Felis catus OX=9685 GN=MTNR1B PE=3 SV=4	Felis catus	380	1567	81.3	88.7	0
42	TR	G1SUP2	Melatonin receptor 1B OS=Oryctolagus cuniculus OX=9986 GN=MTNR1B PE=3 SV=2	Oryctolagus cuniculus	362	1566	83.9	88.1	0
43	TR	A0A9W3FEL9	Melatonin receptor type 1B OS=Camelus bactrianus OX=9837 GN=MTNR1B PE=3 SV=1	Camelus bactrianus	364	1565	81.2	88.9	0
44	TR	A0A8B7T0U0	Melatonin receptor type 1B OS=Hipposideros armiger OX=186990 GN=MTNR1B PE=3 SV=1	Hipposideros armiger	359	1559	81.2	89.9	0
45	TR	A0A6P6I8W4	LOW QUALITY PROTEIN: melatonin receptor type 1B OS=Puma concolor OX=9696 GN=MTNR1B PE=3 SV=1	Puma concolor	473	1558	82.6	89.6	0
46	TR	G3U6B1	Melatonin receptor 1B OS=Loxodonta africana OX=9785 GN=MTNR1B PE=3 SV=1	Loxodonta africana	365	1553	81.6	87.7	0
47	TR	A0A7J7VJ04	Melatonin receptor 1B OS=Myotis myotis OX=51298 GN=mMyoMyo1_013464 PE=3 SV=1	Myotis myotis	365	1550	81.1	87.9	0
48	TR	A0A8C3VT84	Melatonin receptor 1B OS=Catagonus wagneri OX=51154 GN=MTNR1B PE=3 SV=1	Catagonus wagneri	364	1545	80.2	88.2	0
49	TR	H0VHC1	Melatonin receptor 1B OS=Cavia porcellus OX=10141 GN=MTNR1B PE=3 SV=2	Cavia porcellus	366	1541	80.8	88.3	0
50	TR	A0A7J8ET96	Melatonin receptor 1B OS=Molossus molossus OX=27622 GN=HJG59_012607 PE=3 SV=1	Molossus molossus	380	1541	80	87.9	0
51	TR	A0A8C9QMO9	G-protein coupled receptors family 1 profile domain-containing protein OS=Spermophilus dauricus OX=99837 PE=3 SV=1	Spermophilus dauricus	364	1540	81.1	87.7	0
52	TR	A0A7J7X0K5	Melatonin receptor 1B OS=Pipistrellus kuhlii OX=59472 GN=mPipKuh1_011916 PE=3 SV=1	Pipistrellus kuhlii	365	1530	80.2	87.7	0
53	TR	A0A9B0H4X9	Melatonin receptor type 1B OS=Odobenus rosmarus divergens OX=9708 GN=MTNR1B PE=3 SV=1	Odobenus rosmarus divergens	418	1524	81	88	0
54	TR	A0A6J3ABR4	LOW QUALITY PROTEIN: melatonin receptor type 1B OS=Vicugna pacos OX=30538 GN=MTNR1B PE=3 SV=1	Vicugna pacos	388	1516	78.3	85.5	0
55	TR	A0A8C6H9W9	Melatonin receptor 1B OS=Mus spicilegus OX=10103 PE=3 SV=1	Mus spicilegus	364	1501	81.8	86.9	0
56	TR	A0A2K5CCY7	Melatonin receptor 1B OS=Aotus nancymaae OX=37293 GN=MTNR1B PE=3 SV=1	Aotus nancymaae	347	1497	81.4	85.3	0
57	TR	Q3SXF8	Melatonin receptor 1B OS=Mus musculus OX=10090 GN=Mtnr1b PE=2 SV=1	Mus musculus	364	1495	80.9	86.3	0
58	TR	A0A6I9M6I4	Melatonin receptor 1B OS=Peromyscus maniculatus bairdii OX=230844 GN=Mtnr1b PE=3 SV=1	Peromyscus maniculatus bairdii	364	1495	81.1	86.7	0
59	TR	A0A673T838	Melatonin receptor 1B OS=Suricata suricatta OX=37032 PE=3 SV=1	Suricata suricatta	363	1494	79.1	85.7	0
60	SP	Q8CIQ6	Melatonin receptor type 1B OS=Mus musculus OX=10090 GN=Mtnr1b PE=2 SV=1	Mus musculus	364	1489	80.6	86	0
61	TR	A0A8C2VLW4	Melatonin receptor 1B OS=Chinchilla lanigera OX=34839 GN=MTNR1B PE=3 SV=1	Chinchilla lanigera	354	1486	85.8	93.2	0
62	TR	A0A6P3FQJ2	Melatonin receptor type 1B OS=Octodon degus OX=10160 GN=Mtnr1b PE=3 SV=1	Octodon degus	376	1480	80.7	86.4	0
63	TR	A0A6J3JB15	LOW QUALITY PROTEIN: melatonin receptor type 1B OS=Sapajus apella OX=9515 GN=MTNR1B PE=4 SV=1	Sapajus apella	356	1471	82.5	86.7	0
64	SP	P49287	Melatonin receptor type 1B OS=Rattus norvegicus OX=10116 GN=Mtnr1b PE=2 SV=2	Rattus norvegicus	364	1469	80.3	84.9	0
65	TR	A0A3Q7QT12	Melatonin receptor type 1B OS=Callorhinus ursinus OX=34884 GN=MTNR1B PE=4 SV=1	Callorhinus ursinus	461	1465	78.4	86.3	0
66	TR	A0A1A6GUB3	G-protein coupled receptors family 1 profile domain-containing protein OS=Neotoma lepida OX=56216 GN=A6R68_01541 PE=3 SV=1	Neotoma lepida	364	1460	80.1	85.5	0
67	TR	A0A3Q1MJK4	Melatonin receptor 1B OS=Bos taurus OX=9913 GN=MTNR1B PE=3 SV=1	Bos taurus	376	1457	77.8	87.5	0
68	TR	A0A2Y9KDQ2	Melatonin receptor type 1B OS=Enhydra lutris kenyoni OX=391180 GN=LOC111154372 PE=3 SV=1	Enhydra lutris kenyoni	411	1455	77.1	85.1	0
69	TR	A0A4W2BLT7	G-protein coupled receptors family 1 profile domain-containing protein OS=Bos indicus x Bos taurus OX=30522 PE=3 SV=1	Bos indicus x Bos taurus	365	1453	77.5	87.2	0
70	TR	A0A452FBZ9	Melatonin receptor 1B OS=Capra hircus OX=9925 GN=MTNR1B PE=3 SV=1	Capra hircus	376	1445	77.2	86.9	0
71	TR	A0A8C6FQN2	Melatonin receptor 1B OS=Moschus moschiferus OX=68415 GN=MTNR1B PE=3 SV=1	Moschus moschiferus	362	1444	76.9	86.9	0
72	TR	W5NY63	Melatonin receptor 1B OS=Ovis aries OX=9940 GN=MTNR1B PE=3 SV=1	Ovis aries	351	1444	76.9	86.3	0
73	TR	A0AAD4TNF9	G-protein coupled receptors family 1 profile domain-containing protein OS=Ovis ammon polii OX=230172 GN=MG293_019627 PE=3 SV=1	Ovis ammon polii	376	1443	76.9	86.3	0
74	TR	A0A5N3XQY3	G-protein coupled receptors family 1 profile domain-containing protein OS=Muntiacus reevesi OX=9886 GN=FD755_010804 PE=3 SV=1	Muntiacus reevesi	376	1438	76.9	86	0
75	TR	A0A8B9XRS6	Melatonin receptor 1B OS=Bos mutus grunniens OX=30521 GN=MTNR1B PE=3 SV=1	Bos mutus grunniens	363	1432	76.9	86.6	0
76	TR	A0A6J0WBN6	Melatonin receptor type 1B OS=Odocoileus virginianus texanus OX=9880 GN=MTNR1B PE=3 SV=1	Odocoileus virginianus texanus	376	1428	76.1	85.8	0
77	TR	A0A1S3G6G9	Melatonin receptor type 1B OS=Dipodomys ordii OX=10020 GN=Mtnr1b PE=3 SV=1	Dipodomys ordii	367	1426	79.1	86.3	0
78	TR	A0A091DFI9	Melatonin receptor type 1B OS=Fukomys damarensis OX=885580 GN=H920_09371 PE=3 SV=1	Fukomys damarensis	331	1425	80.9	88.8	0
79	TR	A0A2Y9G798	LOW QUALITY PROTEIN: melatonin receptor type 1B OS=Neomonachus schauinslandi OX=29088 GN=MTNR1B PE=4 SV=1	Neomonachus schauinslandi	353	1423	77	84.3	0
80	TR	A0A671FRW6	Melatonin receptor 1B OS=Rhinolophus ferrumequinum OX=59479 GN=MTNR1B PE=3 SV=1	Rhinolophus ferrumequinum	362	1422	75.8	84.6	0
81	TR	A0A2Y9ES79	LOW QUALITY PROTEIN: melatonin receptor type 1B OS=Physeter macrocephalus OX=9755 GN=MTNR1B PE=3 SV=1	Physeter macrocephalus	373	1420	76.4	84.6	0
82	TR	A0A2U3XNH6	LOW QUALITY PROTEIN: melatonin receptor type 1B OS=Leptonychotes weddellii OX=9713 GN=MTNR1B PE=4 SV=1	Leptonychotes weddellii	358	1406	75.6	83	0
83	TR	A0A452C6R9	LOW QUALITY PROTEIN: melatonin receptor type 1B OS=Balaenoptera acutorostrata scammoni OX=310752 GN=MTNR1B PE=3 SV=1	Balaenoptera acutorostrata scammoni	535	1403	75.3	84.2	0
84	TR	A0A6P5BB91	Melatonin receptor type 1B-like OS=Bos indicus OX=9915 GN=LOC109553979 PE=3 SV=1	Bos indicus	500	1386	74.9	84.6	0
85	TR	A0A8U0N0N3	LOW QUALITY PROTEIN: melatonin receptor type 1B OS=Mustela putorius furo OX=9669 GN=MTNR1B PE=4 SV=1	Mustela putorius furo	357	1384	74.1	82.7	0
86	TR	A0A2Y9M5V6	LOW QUALITY PROTEIN: melatonin receptor type 1B OS=Delphinapterus leucas OX=9749 GN=MTNR1B PE=4 SV=1	Delphinapterus leucas	374	1370	73.7	83.6	0
87	TR	A0A8C7AH26	Melatonin receptor 1B OS=Neovison vison OX=452646 GN=MTNR1B PE=3 SV=1	Neovison vison	339	1343	73.6	81.2	0
88	TR	A0A8C0KW83	Melatonin receptor 1B OS=Canis lupus dingo OX=286419 GN=MTNR1B PE=3 SV=1	Canis lupus dingo	347	1340	81.1	87.9	0
89	TR	A0A8B8Y5S3	LOW QUALITY PROTEIN: melatonin receptor type 1B OS=Balaenoptera musculus OX=9771 GN=MTNR1B PE=3 SV=1	Balaenoptera musculus	400	1339	76.3	85.4	0
90	TR	A0A8J6GU08	Melatonin receptor type 1B OS=Microtus ochrogaster OX=79684 GN=LTLLF_125185 PE=3 SV=1	Microtus ochrogaster	364	1337	73.7	82.6	0
91	TR	A0A341ABW4	LOW QUALITY PROTEIN: melatonin receptor type 1B OS=Neophocaena asiaeorientalis asiaeorientalis OX=1706337 GN=MTNR1B PE=4 SV=1	Neophocaena asiaeorientalis asiaeorientalis	376	1331	72.8	82	0
92	TR	A0A340WRM7	LOW QUALITY PROTEIN: melatonin receptor type 1B OS=Lipotes vexillifer OX=118797 GN=MTNR1B PE=3 SV=1	Lipotes vexillifer	363	1331	73.4	83.5	0
93	TR	A0A8C8SCJ7	Melatonin receptor 1B OS=Pelusios castaneus OX=367368 PE=3 SV=1	Pelusios castaneus	359	1323	69.9	82	0
94	TR	A0A8C4VTS0	Melatonin receptor 1B OS=Gopherus evgoodei OX=1825980 GN=MTNR1B PE=3 SV=1	Gopherus evgoodei	358	1317	69.3	81.3	5.5e-180
95	TR	A0A674K988	Melatonin receptor 1B OS=Terrapene triunguis OX=2587831 GN=MTNR1B PE=3 SV=1	Terrapene triunguis	359	1315	69.6	81.3	1.1e-179
96	TR	A0A7K8HYJ1	MTR1B protein (Fragment) OS=Aleadryas rufinucha OX=461220 GN=Mtr1b PE=3 SV=1	Aleadryas rufinucha	362	1315	69	81.6	1.3e-179
97	TR	A0A8C3S6D2	G-protein coupled receptors family 1 profile domain-containing protein OS=Chelydra serpentina OX=8475 PE=3 SV=1	Chelydra serpentina	360	1313	69	80.7	2.4e-179
98	TR	A0A1V4L0D5	Melatonin receptor type 1B OS=Patagioenas fasciata monilis OX=372326 GN=AV530_011159 PE=3 SV=1	Patagioenas fasciata monilis	359	1312	68.8	81.2	3.3e-179
99	TR	A0A7K5YC21	MTR1B protein (Fragment) OS=Pterocles burchelli OX=2585816 GN=Mtr1b PE=3 SV=1	Pterocles burchelli	359	1312	69	81	3.3e-179
100	TR	A0A8D0H9I5	Melatonin receptor 1B OS=Sphenodon punctatus OX=8508 GN=MTNR1B PE=3 SV=1	Sphenodon punctatus	361	1312	68.6	81.1	3.5e-179
101	TR	A0A7L3Q3W7	MTR1B protein (Fragment) OS=Cettia cetti OX=68486 GN=Mtr1b PE=3 SV=1	Cettia cetti	361	1311	69.3	81.8	5e-179
102	TR	A0A7K7V7B6	MTR1B protein (Fragment) OS=Eudromia elegans OX=8805 GN=Mtr1b PE=3 SV=1	Eudromia elegans	361	1311	69.3	81.4	5e-179
103	TR	A0A7K9KMZ7	MTR1B protein (Fragment) OS=Rhabdornis inornatus OX=237438 GN=Mtr1b PE=3 SV=1	Rhabdornis inornatus	361	1311	69	81.8	5e-179
104	TR	A0A7K6IUE0	MTR1B protein (Fragment) OS=Machaerirhynchus nigripectus OX=1160894 GN=Mtr1b PE=3 SV=1	Machaerirhynchus nigripectus	361	1311	69	81.6	5e-179
105	TR	A0A8C0ISL4	Melatonin receptor 1B OS=Chelonoidis abingdonii OX=106734 GN=MTNR1B PE=3 SV=1	Chelonoidis abingdonii	359	1310	68.4	80.2	6.6e-179
106	TR	A0A7L1LSC0	MTR1B protein (Fragment) OS=Bombycilla garrulus OX=125297 GN=Mtr1b PE=3 SV=1	Bombycilla garrulus	360	1310	69.3	81.6	6.8e-179
107	TR	A0A7K7Z3D6	MTR1B protein (Fragment) OS=Thryothorus ludovicianus OX=74200 GN=Mtr1b PE=3 SV=1	Thryothorus ludovicianus	360	1310	69.3	81.8	6.8e-179
108	TR	A0A7K9GYW0	MTR1B protein (Fragment) OS=Loxia leucoptera OX=96539 GN=Mtr1b PE=3 SV=1	Loxia leucoptera	361	1310	69.3	81.6	7.1e-179
109	TR	A0A7K7I5P6	MTR1B protein (Fragment) OS=Loxia curvirostra OX=64802 GN=Mtr1b PE=3 SV=1	Loxia curvirostra	361	1310	69.3	81.6	7.1e-179
110	TR	A0A7K7QG86	MTR1B protein (Fragment) OS=Poecile atricapillus OX=48891 GN=Mtr1b PE=3 SV=1	Poecile atricapillus	361	1310	69.3	81.6	7.1e-179
111	TR	A0A7L3H3S1	MTR1B protein (Fragment) OS=Buphagus erythrorhynchus OX=245048 GN=Mtr1b PE=3 SV=1	Buphagus erythrorhynchus	361	1310	69	81.8	7.1e-179
112	TR	A0A7L1B688	MTR1B protein (Fragment) OS=Gymnorhina tibicen OX=9132 GN=Mtr1b PE=3 SV=1	Gymnorhina tibicen	361	1310	69.3	81.6	7.1e-179
113	TR	A0A7L3PGQ9	MTR1B protein (Fragment) OS=Xiphorhynchus elegans OX=269412 GN=Mtr1b PE=3 SV=1	Xiphorhynchus elegans	361	1309	69.3	81.6	1e-178
114	TR	A0A7K6XH49	MTR1B protein (Fragment) OS=Promerops cafer OX=254652 GN=Mtr1b PE=3 SV=1	Promerops cafer	361	1309	69	81.6	1e-178
115	TR	A0A851NDZ9	MTR1B protein (Fragment) OS=Campylorhamphus procurvoides OX=190295 GN=Mtr1b PE=3 SV=1	Campylorhamphus procurvoides	361	1309	69.3	81.6	1e-178
116	TR	A0A850UKG6	MTR1B protein (Fragment) OS=Chloropsis hardwickii OX=667144 GN=Mtr1b PE=3 SV=1	Chloropsis hardwickii	361	1309	69	81.6	1e-178
117	TR	A0A7L4G8E0	MTR1B protein (Fragment) OS=Pampusana beccarii OX=2953425 GN=Mtr1b PE=3 SV=1	Pampusana beccarii	359	1308	68.5	81	1.3e-178

118	TR	A0A7K6SGG9	MTR1B protein (Fragment) OS=Caloenas nicobarica OX=187106 GN=Mtr1b PE=3 SV=1	Caloenas nicobarica	359	1308	68.5	81	1.3e-178
119	TR	A0A7K7P4I9	MTR1B protein (Fragment) OS=Acrocephalus arundinaceus OX=39621 GN=Mtr1b PE=3 SV=1	Acrocephalus arundinaceus	361	1308	69	81.8	1.4e-178
120	TR	A0A7K6DQZ0	MTR1B protein (Fragment) OS=Origma solitaria OX=720586 GN=Mtr1b PE=3 SV=1	Origma solitaria	361	1308	69.3	81.6	1.4e-178
121	TR	A0A7L0Y328	MTR1B protein (Fragment) OS=Ploceus nigricollis OX=441696 GN=Mtr1b PE=3 SV=1	Ploceus nigricollis	361	1308	69.3	81.6	1.4e-178
122	TR	A0A852HIM3	MTR1B protein (Fragment) OS=Nicator chloris OX=237433 GN=Mtr1b PE=3 SV=1	Nicator chloris	361	1308	69	81.6	1.4e-178
123	TR	A0A7L0X122	MTR1B protein (Fragment) OS=Tyrannus savana OX=137541 GN=Mtr1b PE=3 SV=1	Tyrannus savana	361	1308	69	82.1	1.4e-178
124	TR	A0AA97MTK6	MTR1B protein (Fragment) OS=Menura novaehollandiae OX=47692 GN=Mtr1b PE=3 SV=1	Menura novaehollandiae	361	1308	69	81.8	1.4e-178
125	TR	A0A7K4M197	MTR1B protein (Fragment) OS=Crypturellus undulatus OX=48396 GN=Mtr1b PE=3 SV=1	Crypturellus undulatus	362	1308	68.7	80.8	1.5e-178
126	TR	A0A7K9TAA4	MTR1B protein (Fragment) OS=Galbula dea OX=1109041 GN=Mtr1b PE=3 SV=1	Galbula dea	359	1307	69	80.4	1.9e-178
127	TR	A0A7L1EGD8	MTR1B protein (Fragment) OS=Oenanthe oenanthe OX=279966 GN=Mtr1b PE=3 SV=1	Oenanthe oenanthe	360	1307	69	81.8	2e-178
128	TR	U3JBY1	Melatonin receptor 1B OS=Ficedula albicollis OX=59894 GN=MTNR1B PE=3 SV=1	Ficedula albicollis	360	1307	69	81.8	2e-178
129	TR	A0A7K8GP77	MTR1B protein (Fragment) OS=Orthonyx spaldingii OX=38397 GN=Mtr1b PE=3 SV=1	Orthonyx spaldingii	361	1307	69.6	81.8	2e-178
130	TR	A0A7L2QS19	MTR1B protein (Fragment) OS=Oxylabes madagascariensis OX=98144 GN=Mtr1b PE=3 SV=1	Oxylabes madagascariensis	361	1307	69	81.6	2e-178
131	TR	A0A7K4WK21	MTR1B protein (Fragment) OS=Tachuris rubrigastra OX=495162 GN=Mtr1b PE=3 SV=1	Tachuris rubrigastra	361	1307	69	81.8	2e-178
132	TR	A0A7L4LLU7	MTR1B protein (Fragment) OS=Callaeas wilsoni OX=1347786 GN=Mtr1b PE=3 SV=1	Callaeas wilsoni	361	1307	69	81.6	2e-178
133	TR	A0A7K8QD77	MTR1B protein (Fragment) OS=Smithornis capensis OX=363769 GN=Mtr1b PE=3 SV=1	Smithornis capensis	361	1307	69	81.8	2e-178
134	TR	A0A7L2D9W4	MTR1B protein (Fragment) OS=Catharus fuscescens OX=159581 GN=Mtr1b PE=3 SV=1	Catharus fuscescens	361	1307	68.7	81.6	2e-178
135	TR	A0A7K8ZM90	MTR1B protein (Fragment) OS=Grallaria varia OX=117165 GN=Mtr1b PE=3 SV=1	Grallaria varia	361	1307	69.3	81.6	2e-178
136	TR	A0A7K5Q8H5	MTR1B protein (Fragment) OS=Prunella himalayana OX=670356 GN=Mtr1b PE=3 SV=1	Prunella himalayana	361	1307	69	81.6	2e-178
137	TR	A0A7K7GFZ1	MTR1B protein (Fragment) OS=Erithacus rubecula OX=37610 GN=Mtr1b PE=3 SV=1	Erithacus rubecula	361	1307	69	81.6	2e-178
138	TR	A0A7L3DYL5	MTR1B protein (Fragment) OS=Chaetops frenatus OX=221966 GN=Mtr1b PE=3 SV=1	Chaetops frenatus	361	1307	69	81.6	2e-178
139	TR	A0A7K5LG81	MTR1B protein (Fragment) OS=Vireo altiloquus OX=34956 GN=Mtr1b PE=3 SV=1	Vireo altiloquus	361	1307	69	81.6	2e-178
140	TR	A0A7K5UM30	MTR1B protein (Fragment) OS=Cephalopterus ornatus OX=114276 GN=Mtr1b PE=3 SV=1	Cephalopterus ornatus	361	1307	69.3	81.6	2e-178
141	TR	A0A7K5PDQ8	MTR1B protein (Fragment) OS=Erythrocercus mccallii OX=107208 GN=Mtr1b PE=3 SV=1	Erythrocercus mccallii	361	1307	69	81.6	2e-178
142	TR	A0A7K7SNB1	MTR1B protein (Fragment) OS=Sapayoa aenigma OX=239371 GN=Mtr1b PE=3 SV=1	Sapayoa aenigma	361	1307	69	81.8	2e-178
143	TR	A0A7K5K275	MTR1B protein (Fragment) OS=Mionectes macconnelli OX=254557 GN=Mtr1b PE=3 SV=1	Mionectes macconnelli	361	1307	69.6	81.5	2e-178
144	TR	A0A7K6KC80	MTR1B protein (Fragment) OS=Oreocharis arfaki OX=979223 GN=Mtr1b PE=3 SV=1	Oreocharis arfaki	361	1307	69	81.6	2e-178
145	TR	A0A7K7EN38	MTR1B protein (Fragment) OS=Sylvia atricapilla OX=48155 GN=Mtr1b PE=3 SV=1	Sylvia atricapilla	361	1307	69	81.6	2e-178
146	TR	A0A7K6MR73	MTR1B protein (Fragment) OS=Panurus biarmicus OX=181101 GN=Mtr1b PE=3 SV=1	Panurus biarmicus	361	1307	69	81.6	2e-178
147	TR	A0A7L2T6B4	MTR1B protein (Fragment) OS=Pomatostomus ruficeps OX=9176 GN=Mtr1b PE=3 SV=1	Pomatostomus ruficeps	361	1307	69	81.6	2e-178
148	TR	A0A7K8IJ33	MTR1B protein (Fragment) OS=Chaetorhynchus papuensis OX=254446 GN=Mtr1b PE=3 SV=1	Chaetorhynchus papuensis	361	1307	69	81.6	2e-178
149	TR	A0A7K7C4G6	MTR1B protein (Fragment) OS=Aphelocoma coerulescens OX=39617 GN=Mtr1b PE=3 SV=1	Aphelocoma coerulescens	361	1307	69	81.6	2e-178
150	TR	A0A851UAP4	MTR1B protein (Fragment) OS=Elachura formosa OX=1463973 GN=Mtr1b PE=3 SV=1	Elachura formosa	361	1307	69.3	81.6	2e-178
151	TR	A0A7L2N2T8	MTR1B protein (Fragment) OS=Rhadina sibilatrix OX=2585818 GN=Mtr1b PE=3 SV=1	Rhadina sibilatrix	361	1307	69	81.6	2e-178
152	TR	A0A851VQK5	MTR1B protein (Fragment) OS=Copsychus sechellarum OX=797021 GN=Mtr1b PE=3 SV=1	Copsychus sechellarum	361	1307	69	81.6	2e-178
153	TR	A0A7L0J894	MTR1B protein (Fragment) OS=Piprites chloris OX=114369 GN=Mtr1b PE=3 SV=1	Piprites chloris	361	1307	69	81.8	2e-178
154	TR	A0A7K9K3P4	MTR1B protein (Fragment) OS=Dicaeum eximium OX=667154 GN=Mtr1b PE=3 SV=1	Dicaeum eximium	361	1307	69	81.6	2e-178
155	TR	A0A8C3TM41	Melatonin receptor 1B OS=Catharus ustulatus OX=91951 PE=3 SV=1	Catharus ustulatus	361	1307	68.7	81.6	2e-178
156	TR	A0A851RLL6	MTR1B protein (Fragment) OS=Tychaedon coryphoeus OX=614051 GN=Mtr1b PE=3 SV=1	Tychaedon coryphoeus	361	1307	69	81.6	2e-178
157	TR	A0A7L4IYQ1	MTR1B protein (Fragment) OS=Pomatorhinus ruficollis OX=932028 GN=Mtr1b PE=3 SV=1	Pomatorhinus ruficollis	361	1307	69	81.6	2e-178
158	TR	A0A7K4KAY6	MTR1B protein (Fragment) OS=Crypturellus soui OX=458187 GN=Mtr1b PE=3 SV=1	Crypturellus soui	362	1307	68.7	80.8	2.1e-178
159	TR	A0A8C2SW82	Melatonin receptor 1B OS=Coturnix japonica OX=93934 GN=MTNR1B PE=3 SV=1	Coturnix japonica	363	1307	68.8	80.7	2.2e-178
160	TR	A0A7L3ZB38	MTR1B protein (Fragment) OS=Fregetta grallaria OX=79628 GN=Mtr1b PE=3 SV=1	Fregetta grallaria	359	1306	68.8	80.7	2.7e-178
161	TR	A0A8C5TKG6	Melatonin receptor 1B OS=Malurus cyaneus samueli OX=2593467 PE=3 SV=1	Malurus cyaneus samueli	361	1306	69	81.6	2.9e-178
162	TR	A0A7L2A2R5	MTR1B protein (Fragment) OS=Leiothrix lutea OX=36275 GN=Mtr1b PE=3 SV=1	Leiothrix lutea	361	1306	68.7	81.8	2.9e-178
163	TR	A0A7K4R778	MTR1B protein (Fragment) OS=Neopipo cinnamomea OX=456388 GN=Mtr1b PE=3 SV=1	Neopipo cinnamomea	361	1306	69	81.6	2.9e-178
164	TR	A0A6J2J4H0	Melatonin receptor type 1B isoform X1 OS=Pipra filicauda OX=649802 GN=MTNR1B PE=3 SV=1	Pipra filicauda	361	1306	69	81.6	2.9e-178
165	TR	A0A6J0HKM1	Melatonin receptor type 1B OS=Lepidothrix coronata OX=321398 GN=LOC108499599 PE=3 SV=1	Lepidothrix coronata	361	1306	69	81.6	2.9e-178
166	TR	A0A7L1RH68	MTR1B protein (Fragment) OS=Helopsaltes ochotensis OX=3150915 GN=Mtr1b PE=3 SV=1	Helopsaltes ochotensis	361	1306	68.7	81.3	2.9e-178
167	TR	A0A851KY05	MTR1B protein (Fragment) OS=Vidua chalybeata OX=81927 GN=Mtr1b PE=3 SV=1	Vidua chalybeata	361	1306	68.7	81.6	2.9e-178
168	TR	A0A7K8LRA0	MTR1B protein (Fragment) OS=Ptilorrhoa leucosticta OX=449384 GN=Mtr1b PE=3 SV=1	Ptilorrhoa leucosticta	361	1306	68.7	81.3	2.9e-178
169	TR	A0A851C6B5	MTR1B protein (Fragment) OS=Calyptomena viridis OX=135972 GN=Mtr1b PE=3 SV=1	Calyptomena viridis	361	1306	69	81.6	2.9e-178
170	TR	A0A7L2CH29	MTR1B protein (Fragment) OS=Alaudala cheleensis OX=670337 GN=Mtr1b PE=3 SV=1	Alaudala cheleensis	364	1306	68.6	81.1	3.2e-178
171	TR	A0A7L3B420	MTR1B protein (Fragment) OS=Syrrhaptes paradoxus OX=302527 GN=Mtr1b PE=3 SV=1	Syrrhaptes paradoxus	359	1305	68.8	80.7	3.8e-178
172	TR	A0A7K4RZW6	MTR1B protein (Fragment) OS=Columbina picui OX=115618 GN=Mtr1b PE=3 SV=1	Columbina picui	359	1305	68.5	81	3.8e-178
173	TR	A0A8C3I3P7	Melatonin receptor 1B OS=Chrysemys picta bellii OX=8478 GN=MTNR1B PE=3 SV=1	Chrysemys picta bellii	359	1305	68.4	79.9	3.8e-178
174	TR	A0A7K8YGN5	MTR1B protein (Fragment) OS=Sakesphorus luctuosus OX=419690 GN=Mtr1b PE=3 SV=1	Sakesphorus luctuosus	361	1305	69	81.3	4.1e-178
175	TR	A0A7K6EM16	MTR1B protein (Fragment) OS=Grantiella picta OX=266360 GN=Mtr1b PE=3 SV=1	Grantiella picta	361	1305	69	81.6	4.1e-178
176	TR	A0A7K4XG88	MTR1B protein (Fragment) OS=Regulus satrapa OX=13245 GN=Mtr1b PE=3 SV=1	Regulus satrapa	365	1305	67.8	80.3	4.7e-178
177	TR	A0A852L9G4	MTR1B protein (Fragment) OS=Urocolius indicus OX=458196 GN=Mtr1b PE=3 SV=1	Urocolius indicus	359	1304	68.5	80.7	5.4e-178
178	TR	A0A7K6TPN6	MTR1B protein (Fragment) OS=Aegotheles bennettii OX=48278 GN=Mtr1b PE=3 SV=1	Aegotheles bennettii	359	1304	68.5	80.7	5.4e-178
179	TR	A0A7K7MLX5	MTR1B protein (Fragment) OS=Brachypodius melanocephalos OX=3235156 GN=Mtr1b PE=3 SV=1	Brachypodius melanocephalos	361	1304	68.7	81.6	5.8e-178
180	TR	A0A8C5NTS7	Melatonin receptor 1B OS=Junco hyemalis OX=40217 PE=3 SV=1	Junco hyemalis	361	1304	69.3	81.3	5.8e-178
181	TR	A0A8K1LHY4	G-protein coupled receptors family 1 profile domain-containing protein OS=Zosterops borbonicus OX=364589 GN=HGM15179_012470 PE=3 SV=1	Zosterops borbonicus	361	1304	68.7	81.6	5.8e-178
182	TR	A0A7L2PRW7	MTR1B protein (Fragment) OS=Hypocryptadius cinnamomeus OX=589841 GN=Mtr1b PE=3 SV=1	Hypocryptadius cinnamomeus	361	1304	68.7	81.6	5.8e-178
183	TR	A0A7L0R274	MTR1B protein (Fragment) OS=Setophaga kirtlandii OX=298831 GN=Mtr1b PE=3 SV=1	Setophaga kirtlandii	361	1304	69	81.3	5.8e-178
184	TR	A0A7L0NGR7	MTR1B protein (Fragment) OS=Formicarius rufipectus OX=1118560 GN=Mtr1b PE=3 SV=1	Formicarius rufipectus	361	1304	68.7	81.6	5.8e-178
185	TR	A0A7L1HRM0	MTR1B protein (Fragment) OS=Nycticryphes semicollaris OX=227226 GN=Mtr1b PE=3 SV=1	Nycticryphes semicollaris	363	1304	68.5	81	6.2e-178
186	TR	A0A7K9Q337	MTR1B protein (Fragment) OS=Pachycephala philippinensis OX=449367 GN=Mtr1b PE=3 SV=1	Pachycephala philippinensis	367	1304	68.6	80.7	7.2e-178
187	TR	A0A8C3MBW7	Uncharacterized protein OS=Geospiza parvula OX=87175 GN=MTNR1B PE=3 SV=1	Geospiza parvula	361	1303	69	81.3	8.2e-178
188	TR	A0A7K8REZ6	MTR1B protein (Fragment) OS=Rhodinocichla rosea OX=58203 GN=Mtr1b PE=3 SV=1	Rhodinocichla rosea	361	1303	69	81.3	8.2e-178
189	TR	A0A852CZA1	MTR1B protein (Fragment) OS=Passerina amoena OX=142471 GN=Mtr1b PE=3 SV=1	Passerina amoena	361	1303	69	81.3	8.2e-178
190	TR	A0A7K6A7T3	MTR1B protein (Fragment) OS=Onychorhynchus coronatus OX=360224 GN=Mtr1b PE=3 SV=1	Onychorhynchus coronatus	361	1303	69	81.3	8.2e-178
191	TR	A0A7L3VCZ7	MTR1B protein (Fragment) OS=Molothrus ater OX=84834 GN=Mtr1b PE=3 SV=1	Molothrus ater	361	1303	69	81.3	8.2e-178
192	TR	A0A7K9N6H9	MTR1B protein (Fragment) OS=Edolisoma coerulescens OX=2585810 GN=Mtr1b PE=3 SV=1	Edolisoma coerulescens	361	1303	69	81.3	8.2e-178
193	TR	A0A7K7CVS4	MTR1B protein (Fragment) OS=Pheucticus melanocephalus OX=371919 GN=Mtr1b PE=3 SV=1	Pheucticus melanocephalus	361	1303	69	81.3	8.2e-178
194	TR	A0A7K4UWA9	MTR1B protein (Fragment) OS=Emberiza fucata OX=337179 GN=Mtr1b PE=3 SV=1	Emberiza fucata	361	1303	68.7	81.6	8.2e-178
195	TR	A0A7L4F1G8	MTR1B protein (Fragment) OS=Hirundo rustica OX=43150 GN=Mtr1b PE=3 SV=1	Hirundo rustica	361	1303	69	81.3	8.2e-178
196	TR	A0A851WHJ1	MTR1B protein (Fragment) OS=Corvus moneduloides OX=1196302 GN=Mtr1b PE=3 SV=1	Corvus moneduloides	361	1303	69	81.3	8.2e-178
197	TR	A0A7L2Z4M7	MTR1B protein (Fragment) OS=Jacana jacana OX=54508 GN=Mtr1b PE=3 SV=1	Jacana jacana	363	1303	68.6	80.7	8.8e-178
198	TR	A0A6J0VET7	Melatonin receptor type 1B OS=Pogona vitticeps OX=103695 GN=LOC110091096 PE=3 SV=1	Pogona vitticeps	363	1303	69.3	79.8	8.8e-178
199	TR	A0A7K4Y7A5	MTR1B protein (Fragment) OS=Bucorvus abyssinicus OX=153643 GN=Mtr1b PE=3 SV=1	Bucorvus abyssinicus	359	1302	68.5	80.7	1.1e-177
200	TR	A0A7K6H6A5	MTR1B protein (Fragment) OS=Malurus elegans OX=720584 GN=Mtr1b PE=3 SV=1	Malurus elegans	361	1302	69	81.3	1.2e-177
201	TR	A0A7K9DAU6	MTR1B protein (Fragment) OS=Hemiprocne comata OX=243314 GN=Mtr1b PE=3 SV=1	Hemiprocne comata	359	1301	69.3	80.1	1.5e-177
202	TR	A0A7K9I1I0	MTR1B protein (Fragment) OS=Bucco capensis OX=135168 GN=Mtr1b PE=3 SV=1	Bucco capensis	359	1301	68.5	80.7	1.5e-177
203	TR	A0A7K9LYG1	MTR1B protein (Fragment) OS=Oceanodroma tethys OX=79633 GN=Mtr1b PE=3 SV=1	Oceanodroma tethys	359	1301	67.9	80.2	1.5e-177
204	TR	A0A7K9R9Y5	MTR1B protein (Fragment) OS=Irena cyanogastra OX=175120 GN=Mtr1b PE=3 SV=1	Irena cyanogastra	360	1301	68.7	81.6	1.6e-177
205	TR	A0A7K9Z538	MTR1B protein (Fragment) OS=Odontophorus gujanensis OX=886794 GN=Mtr1b PE=3 SV=1	Odontophorus gujanensis	361	1301	69.6	81	1.7e-177
206	TR	A0A7L3IE40	MTR1B protein (Fragment) OS=Pardalotus punctatus OX=254575 GN=Mtr1b PE=3 SV=1	Pardalotus punctatus	361	1301	69	81.3	1.7e-177
207	TR	A0A7L1D4X6	MTR1B protein (Fragment) OS=Serilophus lunatus OX=239386 GN=Mtr1b PE=3 SV=1	Serilophus lunatus	361	1301	68.2	81.8	1.7e-177
208	TR	A0A7K6QFZ6	MTR1B protein (Fragment) OS=Climacteris rufus OX=47695 GN=Mtr1b PE=3 SV=1	Climacteris rufus	361	1301	69	81.3	1.7e-177
209	TR	A0A0A0MQ60	Melatonin receptor 1B OS=Gallus gallus OX=9031 GN=MTNR1B PE=3 SV=1	Gallus gallus	361	1301	69.5	80.5	1.7e-177
210	TR	A0A7K5AXD4	MTR1B protein (Fragment) OS=Furnarius figulus OX=463165 GN=Mtr1b PE=3 SV=1	Furnarius figulus	361	1301	69	81.3	1.7e-177
211	TR	A0A7L0VQ48	MTR1B protein (Fragment) OS=Leptocoma aspasia OX=2585812 GN=Mtr1b PE=3 SV=1	Leptocoma aspasia	361	1300	68.7	81.3	2.3e-177
212	TR	A0A8C6ZQ56	Melatonin receptor 1B OS=Nothoprocta perdicaria OX=30464 GN=MTNR1B PE=3 SV=1	Nothoprocta perdicaria	363	1300	68.2	81.1	2.5e-177
213	TR	A0A7K7AHR0	MTR1B protein (Fragment) OS=Nothoprocta ornata OX=83376 GN=Mtr1b PE=3 SV=1	Nothoprocta ornata	363	1300	68.2	81.1	2.5e-177
214	TR	A0A7L1GFW9	MTR1B protein (Fragment) OS=Indicator maculatus OX=545262 GN=Mtr1b PE=3 SV=1	Indicator maculatus	359	1299	68.5	80.1	3.1e-177
215	TR	A0A8C9F6Q1	Melatonin receptor 1B OS=Pavo cristatus OX=9049 PE=3 SV=1	Pavo cristatus	361	1299	69.2	80.5	3.3e-177
216	TR	A0A8B9F542	Melatonin receptor 1B OS=Amazona collaria OX=241587 PE=3 SV=1	Amazona collaria	362	1299	68.6	79.6	3.5e-177
217	TR	A0A7K6VW26	MTR1B protein (Fragment) OS=Steatornis caripensis OX=48435 GN=Mtr1b PE=3 SV=1	Steatornis caripensis	358	1298	68.3	80.2	4.2e-177
218	TR	A0A8D0FRZ0	Melatonin receptor 1B OS=Strix occidentalis caurina OX=311401 PE=3 SV=1	Strix occidentalis caurina	359	1298	67.6	80.7	4.4e-177
219	TR	A0A8C3RRM3	G-protein coupled receptors family 1 profile domain-containing protein OS=Chelydra serpentina OX=8475 PE=3 SV=1	Chelydra serpentina	359	1298	69	80.7	4.4e-177
220	TR	A0A669PI34	Melatonin receptor 1B OS=Phasianus colchicus OX=9054 PE=3 SV=1	Phasianus colchicus	361	1298	69.2	80.5	4.7e-177
221	TR	A0A226MVA7	G-protein coupled receptors family 1 profile domain-containing protein OS=Callipepla squamata OX=9009 GN=ASZ78_006369 PE=3 SV=1	Callipepla squamata	361	1298	69.3	81	4.7e-177
222	TR	A0AA40L1Z9	MTR1B protein (Fragment) OS=Pygoscelis papua OX=30457 GN=Mtr1b PE=3 SV=1	Pygoscelis papua	359	1297	67.9	80.7	6.2e-177
223	TR	A0A7L0KJE5	MTR1B protein (Fragment) OS=Chauna torquata OX=30388 GN=Mtr1b PE=3 SV=1	Chauna torquata	359	1297	68.5	80.7	6.2e-177
224	TR	A0A850VJN8	MTR1B protein (Fragment) OS=Fregata magnificens OX=37042 GN=Mtr1b PE=3 SV=1	Fregata magnificens	359	1297	68.5	80.4	6.2e-177
225	TR	A0A851Z8N6	MTR1B protein (Fragment) OS=Halcyon senegalensis OX=342381 GN=Mtr1b PE=3 SV=1	Halcyon senegalensis	359	1297	68.5	81	6.2e-177
226	TR	A0A7L0TE59	MTR1B protein (Fragment) OS=Podilymbus podiceps OX=9252 GN=Mtr1b PE=3 SV=1	Podilymbus podiceps	359	1296	68.2	80.4	8.9e-177
227	TR	A0A7L4A0R7	MTR1B protein (Fragment) OS=Circaetus pectoralis OX=321084 GN=Mtr1b PE=3 SV=1	Circaetus pectoralis	359	1296	68.2	80.7	8.9e-177
228	TR	A0A7K9UWZ3	MTR1B protein (Fragment) OS=Anseranas semipalmata OX=8851 GN=Mtr1b PE=3 SV=1	Anseranas semipalmata	359	1296	68.5	80.1	8.9e-177
229	TR	A0A7K8XTM9	MTR1B protein (Fragment) OS=Eubucco bourcierii OX=91767 GN=Mtr1b PE=3 SV=1	Eubucco bourcierii	359	1296	68.5	80.1	8.9e-177
230	TR	A0A7K5HBV6	MTR1B protein (Fragment) OS=Crotophaga sulcirostris OX=33598 GN=Mtr1b PE=3 SV=1	Crotophaga sulcirostris	359	1296	68.2	80.7	8.9e-177
231	TR	A0A7L1A5T1	MTR1B protein (Fragment) OS=Oxyruncus cristatus OX=114331 GN=Mtr1b PE=3 SV=1	Oxyruncus cristatus	360	1296	68.7	81	9.2e-177
232	TR	A0A672TQN9	Melatonin receptor 1B OS=Strigops habroptila OX=2489341 GN=MTNR1B PE=3 SV=1	Strigops habroptila	363	1296	67.5	79.6	1e-176
233	TR	A0A7K9BKW9	MTR1B protein (Fragment) OS=Psilopogon haemacephalus OX=2585815 GN=Mtr1b PE=3 SV=1	Psilopogon haemacephalus	359	1295	68.2	80.1	1.3e-176
234	TR	A0A8J4UMA0	G-protein coupled receptors family 1 profile domain-containing protein (Fragment) OS=Aptenodytes patagonicus OX=9234 GN=FQA23_0004778 PE=3 SV=1	Aptenodytes patagonicus	359	1295	67.3	79.9	1.3e-176
235	TR	A0A7L1IPS4	MTR1B protein (Fragment) OS=Smutsornis africanus OX=240209 GN=Mtr1b PE=3 SV=1	Smutsornis africanus	370	1296	69.6	81.4	1.3e-176

236	TR	A0A852P8E9	MTR1B protein (Fragment) OS=Atrichornis clamosus OX=449594 GN=Mtr1b PE=3 SV=1	Atrichornis clamosus	361	1295	68.4	81	1.4e-176
237	TR	A0A7L0CIS4	MTR1B protein (Fragment) OS=Rostratula benghalensis OX=118793 GN=Mtr1b PE=3 SV=1	Rostratula benghalensis	363	1295	68.2	80.1	1.5e-176
238	TR	A0A8B9Q9R0	Melatonin receptor 1B OS=Apteryx owenii OX=8824 PE=3 SV=1	Apteryx owenii	366	1295	67.3	80.3	1.6e-176
239	TR	A0A8C8B3E8	Melatonin receptor 1B OS=Otus sunia OX=257818 PE=3 SV=1	Otus sunia	359	1294	67	80.4	1.8e-176
240	TR	A0A7K5GA48	MTR1B protein (Fragment) OS=Chunga burmeisteri OX=1352770 GN=Mtr1b PE=3 SV=1	Chunga burmeisteri	359	1294	68.2	80.4	1.8e-176
241	TR	A0A7L1PCG9	MTR1B protein (Fragment) OS=Oriolus oriolus OX=181099 GN=Mtr1b PE=3 SV=1	Oriolus oriolus	361	1294	68.4	81	1.9e-176
242	TR	A0A8D0DPB6	Melatonin receptor 1B OS=Salvator merianae OX=96440 GN=MTNR1B PE=3 SV=1	Salvator merianae	358	1293	68.6	81	2.4e-176
243	TR	A0A7K6SDM5	MTR1B protein (Fragment) OS=Rhynochetos jubatus OX=54386 GN=Mtr1b PE=3 SV=1	Rhynochetos jubatus	359	1293	68.2	80.7	2.5e-176
244	TR	A0A7L0GMX9	MTR1B protein (Fragment) OS=Herpetotheres cachinnans OX=56343 GN=Mtr1b PE=3 SV=1	Herpetotheres cachinnans	359	1293	67.9	80.4	2.5e-176
245	TR	A0A663EHL1	Melatonin receptor 1B OS=Aquila chrysaetos chrysaetos OX=223781 GN=MTNR1B PE=3 SV=1	Aquila chrysaetos chrysaetos	359	1293	68.5	80.4	2.5e-176
246	TR	A0A7K5F508	MTR1B protein (Fragment) OS=Probosciger aterrimus OX=141839 GN=Mtr1b PE=3 SV=1	Probosciger aterrimus	363	1293	68.5	79.6	2.9e-176
247	TR	A0A8C3NP30	Melatonin receptor 1B OS=Cyanoderma ruficeps OX=181631 PE=3 SV=1	Cyanoderma ruficeps	556	1312	69	81.8	3.1e-176
248	TR	A0A7L0BG35	MTR1B protein (Fragment) OS=Spizaetus tyrannus OX=252798 GN=Mtr1b PE=3 SV=1	Spizaetus tyrannus	359	1292	68.2	80.4	3.6e-176
249	TR	A0A8C3JNJ1	Melatonin receptor 1B OS=Calidris pygmaea OX=425635 PE=3 SV=1	Calidris pygmaea	364	1292	67.5	79.8	4.3e-176
250	TR	A0A852I786	MTR1B protein (Fragment) OS=Tricholaema leucomelas OX=240729 GN=Mtr1b PE=3 SV=1	Tricholaema leucomelas	359	1291	68.2	79.8	5.1e-176
